# Iridium-Catalysed *ortho*-Directed Deuterium Labelling of Aromatic Esters—An Experimental and Theoretical Study on Directing Group Chemoselectivity

**DOI:** 10.3390/molecules200711676

**Published:** 2015-06-25

**Authors:** Jennifer Devlin, William. J. Kerr, David M. Lindsay, Timothy J. D. McCabe, Marc Reid, Tell Tuttle

**Affiliations:** Department of Pure & Applied Chemistry, University of Strathclyde, WestCHEM, 295 Cathedral Street, Glasgow G1 1XL, UK; E-Mails: jennifer.devlin.2013@uni.strath.ac.uk (J.D.); david.lindsay@strath.ac.uk (D.M.L.); timothy.mccabe@strath.ac.uk (T.J.D.M.); marc.reid@ed.ac.uk (M.R.); tell.tuttle@strath.ac.uk (T.T.)

**Keywords:** hydrogen isotope exchange, deuterium, iridium, esters, C–H activation, DFT

## Abstract

Herein we report a combined experimental and theoretical study on the deuterium labelling of benzoate ester derivatives, utilizing our developed iridium *N*-heterocyclic carbene/phosphine catalysts. A range of benzoate esters were screened, including derivatives with electron-donating and -withdrawing groups in the *para*- position. The substrate scope, in terms of the alkoxy group, was studied and the nature of the catalyst counter-ion was shown to have a profound effect on the efficiency of isotope exchange. Finally, the observed chemoselectivity was rationalized by rate studies and theoretical calculations, and this insight was applied to the selective labelling of benzoate esters bearing a second directing group.

## 1. Introduction

The ability to incorporate an isotopic label into a biologically-active molecule is of profound importance in the drug discovery process. Such a radioactive “tag” or “label” can be used to provide vital information on a compound’s absorption, distribution, metabolism, excretion, and toxicological (ADMET) properties. As a result of these uses, isotopic labelling is the gold standard method by which early stage drug discovery processes can be optimised.

Research into deuterium (^2^H or D) and tritium (^3^H or T) labelling is more substantial than for other isotopes, and has been developed on a number of fronts over the past 60 years [1,2,3,4,5,6,7,8,9,10]. Further to this, key developments in synthetic strategies and analytical techniques for tritium labelling over the past three decades now makes this the preferred technique for many ADMET studies [5]. In one particularly active branch of such labelling research, hydrogen isotope exchange (HIE) is employed to deliver either deuterium or radioactive tritium to pharmaceutical drug candidates in one synthetic step. As well as circumventing the requirement for isotopically-enriched starting materials in preparing tritiated drug candidates [1,5], HIE can also provide analogous deuterated compounds for use as internal standards for mass spectrometry [11,12], for kinetic isotope studies [13,14], and for the alteration of reaction pathways in total synthesis [15].

**Scheme 1:**
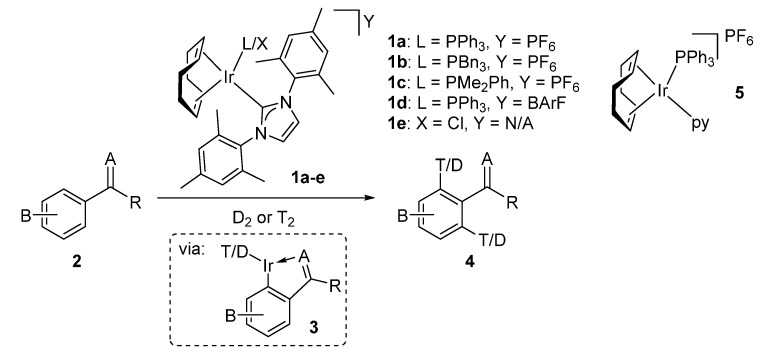
General method for Ir-catalysed *ortho*-HIE via a C–H activation pathway.

Over recent years, research in our laboratory has focused on the development of iridium(I) *N*-heterocyclic carbene (NHC)-ligated precatalysts of the type **1**, and their application in HIE via *ortho*-directed C–H activation protocols (**2**→**4** via **3**, Scheme 1) [6,16,17,18,19,20,21,22]. Despite the growing list of compatible directing groups, we [23] and others [24,25,26,27] have found these developed C–H activation methods less applicable in the labelling of aromatic esters under ambient conditions. Herein, we report the extension of our methodology to encompass the labelling of weakly coordinating benzoate ester derivatives under mild conditions.

## 2. Results and Discussion

### 2.1. Catalyst Screening and Comparisons with Crabtree’s Catalyst

**Scheme 2:**
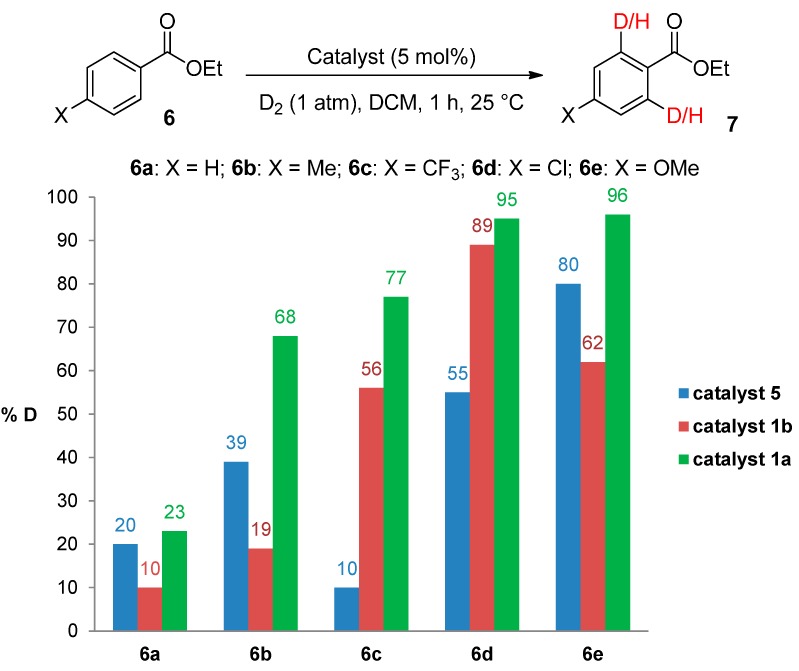
Comparative labelling of ethyl benzoates **6** using catalysts **5**, **1b**, and **1a**.

Until recently, Crabtree’s catalyst, [(COD)Ir(PCy_3_)(py)]PF_6_, **5**, was the most widely applied iridium-based HIE catalyst for labelling applications within an industrial setting [24,28]. As such, any studies which evaluate our developed catalysts in the labelling of aromatic esters should also compare them against the ability of **5** to mediate the same catalytic labelling reactions under identical conditions [19,29]. To this end, and to initiate this research programme, the labelling of a series of *para*-substituted ethyl benzoate derivatives **6** was examined, using our standard labelling protocol (5 mol % [Ir], 1 atm D_2_, 1 h) with Crabtree’s catalyst **5** (Scheme 2, blue bars) and our developed catalyst systems **1b** and **1a** (Scheme 2, red and green bars, respectively). With the exception of the *p*-chloro and *p*-methoxy esters (**6d** and **6e**), Crabtree’s catalyst **5**, proved relatively ineffective in the deuteration of these ester substrates, with incorporations as low as 10% being observed with the electron-withdrawing *p*-CF_3_ ester **6c**. On assessing the larger and more electron-rich variant of our catalyst series, **1b**, a significant improvement in labelling esters **6c** (X = CF_3_) and **6d** (X = Cl) was observed, whereas the other esters **6a**, **6b** and **6e** were labelled less efficiently relative to Crabtree’s catalyst. Only on employing our more Lewis acidic catalyst, **1a**, did we observe the most efficient and encouraging improvement in ester labelling across all examples tested, with the exception of the parent ethyl benzoate **6a**. We hypothesize that the more flexible Lewis acidity of **1a** *vs.* **5** or **1b** partially diminishes the importance of the ester coordination event and negative Hammett σ_p_ values,[30] and simultaneously enhances the effect of positive σ_m_ in relation to a more facile C–H activation event. For example, compare the order of substrate reactivity for catalyst **5** (OMe > Cl > Me > H > CF_3_) *vs.* **1a** (OMe–Cl > CF_3_ > Me > H). In the absence of more detailed reaction monitoring, we acknowledge that the observed results cannot be directly related to the kinetically-derived Hammett values. Nonetheless, the hypothesis remains feasible.

### 2.2. Applicable Substrate Scope with Catalyst **1a**

#### 2.2.1. Para-Substituted Methyl Benzoates

Using the most efficient of the three catalysts tested, catalyst **1a**, we investigated the possibility of labelling methyl esters under the same conditions employed for the ethyl analogues **6** (Table 1). While methyl esters **8d** and **8e** were labelled with high levels of deuterium incorporation, the other methyl esters in the series proved more capricious. Specifically, substrates **8a**, **8b** and **8c** were repeated multiple times, with individual deuterium incorporations ranging from 27% to 52% (see Experimental Section for full details).

**Table 1:** Labelling of methyl benzoates **8** using catalyst **1a**.

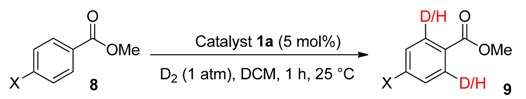

Entry	X	Substrate	%D ^a^
1	H	**8a**	52
2	CH_3_	**8b**	42
3	CF_3_	**8c**	32
4	Cl	**8d**	93
5	OMe	**8e**	89

^a^ %D incorporation is the average of two runs and was determined by ^1^H-NMR spectroscopy.

#### 2.2.2. Scope of *O*-Alkyl Substitution with Electron-Rich Benzoate Esters

Beyond methyl and ethyl benzoates, we also examined the applicability of larger *O*-alkyl ester substituents using our labelling method (Table 2). We pursued this line of enquiry for *p*-methoxybenzoate derivatives only, in order to minimise potential substrate coordination issues associated with the aryl substituent. Whilst the *n*-propyl, 2,2,2-trifluoroethyl, and *tert*-butyl benzoate derivatives **10a**, **10b** and **10c** unfortunately proved to be less applicable, *iso*-propyl and benzyl esters **10d** and **10e** could be labelled with appreciable levels of deuterium incorporation.

**Table 2:** Labelling of electron-rich benzoate esters using catalyst **1a**.

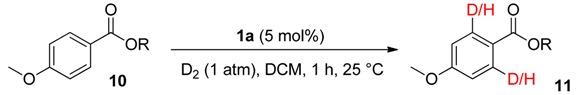

Entry	R	Substrate	%D ^a^
1	*n*-Pr	**10a**	28
2	CH_2_CF_3_	**10b**	8
3	*t*-Bu	**10c**	10
4	*i*-Pr	**10d**	73
5	Bn	**10e**	62

^a^ %D incorporation is the average of two runs and was determined by ^1^H-NMR spectroscopy.

### 2.3. Reaction Optimisation for Efficient Labelling of Challenging Benzoate Ester Substrates

#### 2.3.1. Temperature Effects

**Scheme 3:**
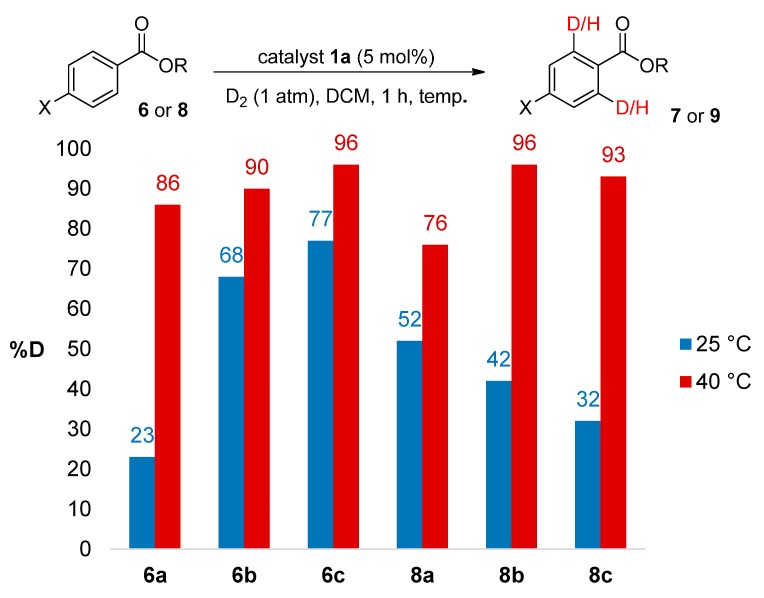
Temperature effects on deuterium labelling of previously problematic benzoate esters.

Due to the application of this hydrogen isotope exchange method in related tritiation chemistries [6], significant effort is usually made to maintain ambient reaction conditions during the optimization of *ortho*-deuteration processes. Having stated this, the use of slightly raised reaction temperatures need not be completely discounted from such investigations. We therefore revisited the labelling of the most challenging methyl and ethyl benzoate derivatives, using a moderately increased reaction temperature of 40 °C (Scheme 3). Pleasingly, dramatic improvements in deuterium incorporation were observed across all substrates examined, **6a**–**c** and **8a**–**c**, whilst the short reaction times were maintained (see Experimental Section 3.4).

#### 2.3.2. Catalyst Counterion Effects

**Scheme 4:**
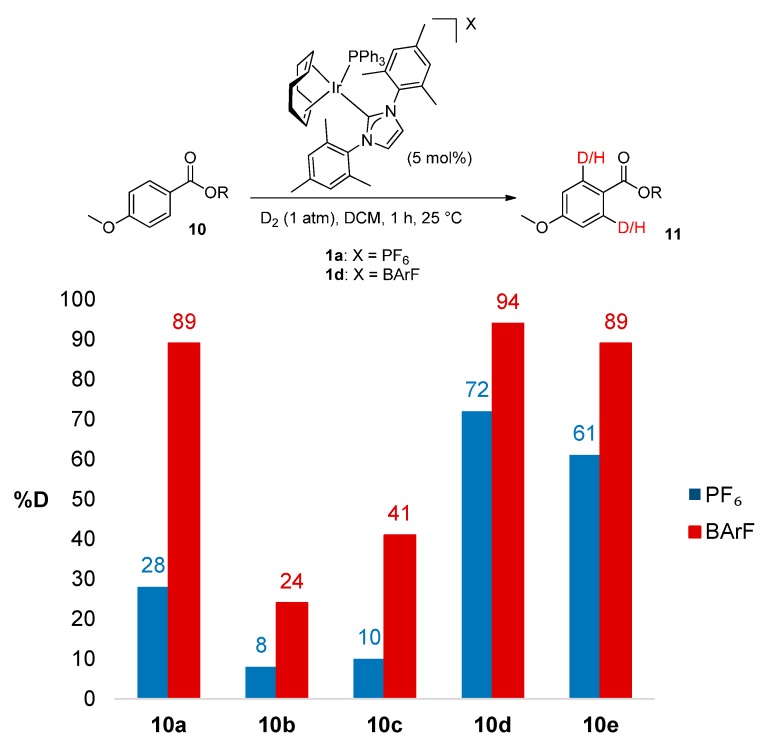
Exploiting anion effects for improved ester labelling.

We recently reported the improved activity and broad-spectrum solubility resulting from replacement of the PF_6_ counterion in catalyst **1a** with tetrakis[bis-3,5-trifluoromethylphenyl]borate, BArF, to give complex **1d** [18]. Applying improved catalyst **1d** to the labelling of larger ester derivatives **10a**–**e** under otherwise identical conditions, significant and more usable levels of deuterium incorporation were observed across all examples (Scheme 4). Importantly, this counter-ion switch demonstrates an alternative means by which ester labelling efficiency can be improved, should ambient temperature conditions be required.

### 2.4. Exploring Chemoselectivity Issues in Ester Labelling

#### 2.4.1. Experimental Observations

**Scheme 5:**
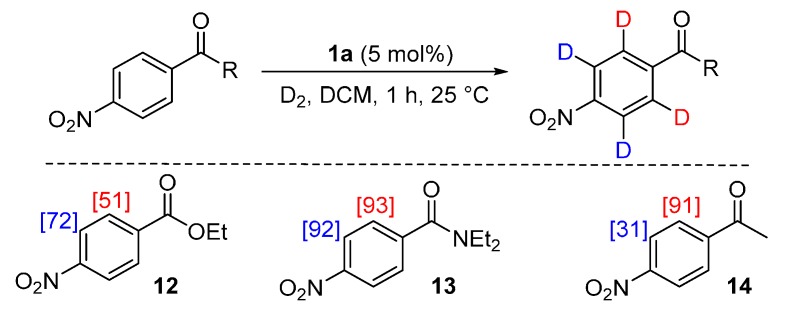
Variation in labelling regioselectivity based on directing group chemoselectivity.

From the outset of our studies, it was clear that the main challenge in labelling aromatic esters would be the weak coordinating ability of this functional group. To understand this issue in more detail, we conducted a series of intramolecular competition studies where multiple potential directing groups can compete for coordination (and subsequent C–H activation) at the iridium centre. To this end, we first investigated the labelling of ethyl *p*-nitrobenzoate, **12**, under the optimised ambient reaction conditions. Interestingly, we observed an approximate 1.4:1 selectivity for labelling via the nitro rather than the ester directing group (Scheme 5). This chemoselectivity was eroded entirely on changing the ester to a tertiary amide in **13** (1:1 nitro:amide), and reversed by replacing the ester with a ketone in **14** (3:1 in favour of the ketone).

**Scheme 6:**
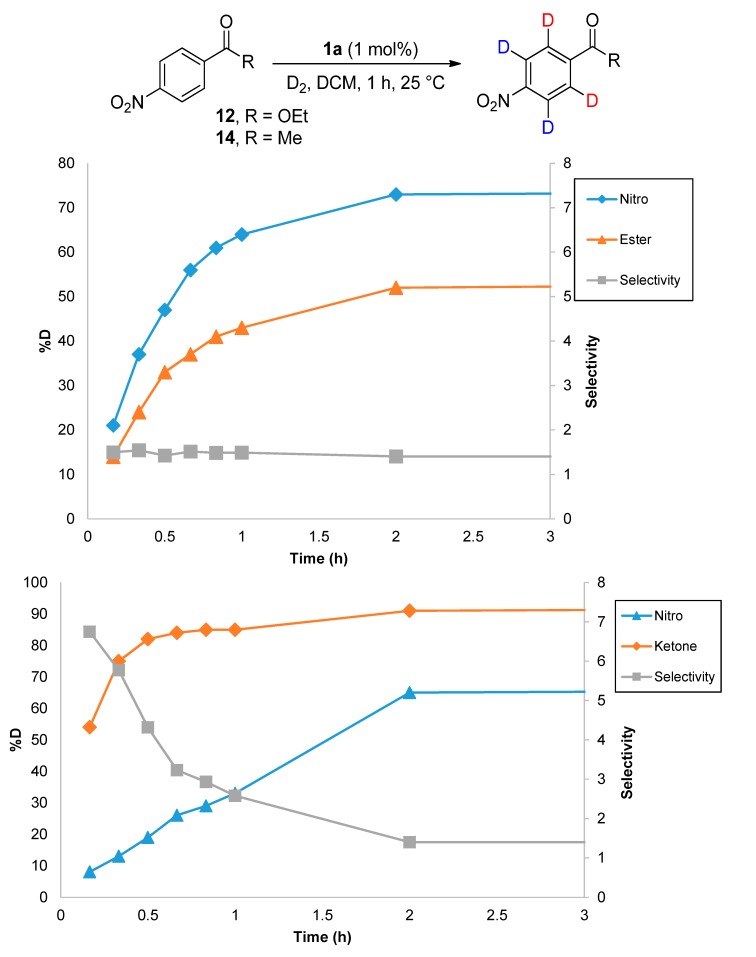
Rate and product selectivity studies for **12** (**top**) and **14** (**bottom**).

Focusing on the extreme substrate cases, with substrates **12** and **14**, 1 mol % of catalyst **1a** was employed to allow reaction rates and labelling selectivities to be monitored over time (Scheme 6). In the case of substrate **12**, and the labelling of the positions *ortho*- to the ester *vs.* the nitro, the difference in reaction rate, and thus product selectivity, remains largely constant throughout the course of the reaction. Conversely, with substrate **14**, the relative rate of labelling *ortho*- to ketone *vs.* nitro is higher at lower conversions, and decreases rapidly over time.

#### 2.4.2. Theoretical Analysis

**Scheme 7:**
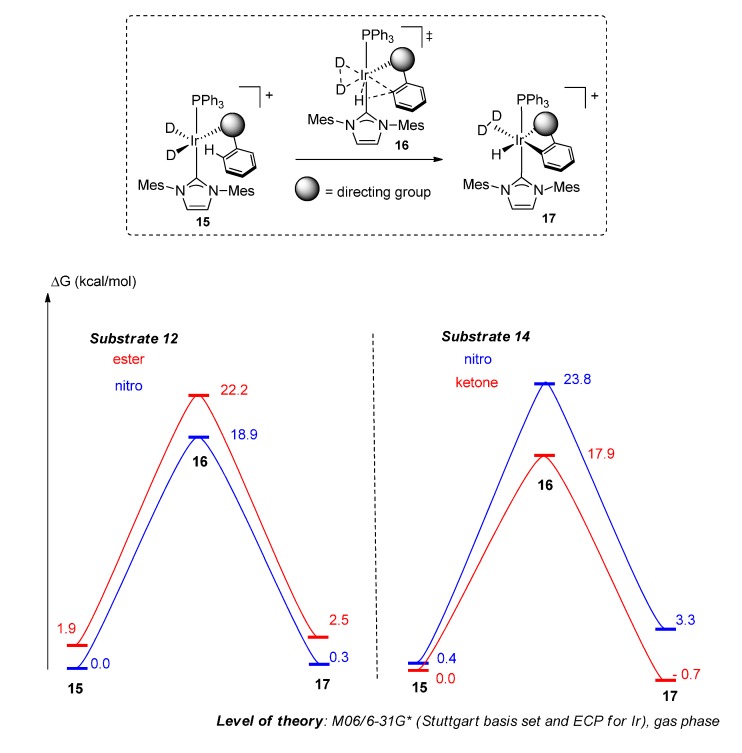
Density functional theory (DFT) analyses on the C–H activation step in labelling substrates **12** and **14** with **1a**.

In previous C–H activation studies, we rationalised observed directing group chemoselectivity using density functional theory (DFT) calculations (see Supplementary Materials for details) to model the relative energies of the binding conformers and subsequent C–H activation pathways [20]. We have now extended this approach to the analysis of the labelling reactions of **12** and **14** (Scheme 7). In agreement with previous findings, we qualitatively predicted that the most stable binding isomer should also be the most reactive. If Curtin-Hammett kinetics are assumed [31], the calculated ΔΔG^‡^ (and thus product selectivity) from equilibrium and activation parameters is predicted to be higher for the ketone in **14** *vs.* the nitro group in **12** (5.5 *vs.* 1.4 kcal/mol, respectively). However, the current model does not account for the barrier to interconversion of binding conformations (ketone to nitro, ester to nitro, and *vice versa* for each case). Considering these points in the context of the experimentally-determined product selectivity *vs.* time (*vide supra*), only substrate **12** (showing little variation in selectivity over time) appears to show rapid equilibrium between the binding isomers. Conversely, the labelling of **14** via the ketone may be interpreted as being faster than the rate of interconversion between binding conformers as well as possessing a lower barrier to C–H activation.

#### 2.4.3. Practical Exploitation of Directing Group Chemoselectivity

**Scheme 8:**
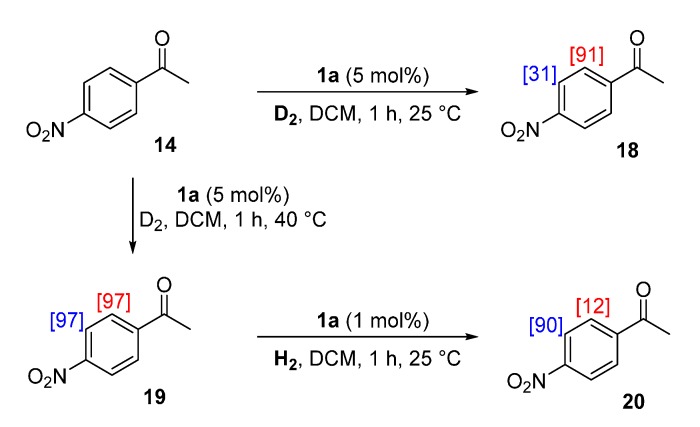
Condition-dependent flexible access to alternatively deuterated forms of **18**.

The fundamental analysis of intramolecular directing group chemoselectivity served to show that observed labelling patterns are, in part, dependent on the relative catalyst binding affinities of each directing group. With this new understanding in hand, we questioned if it would be possible to control the direction of labelling using the inherent reactivity of a given multi-functional substrate. Pleasingly, using substrate **14** as a proof-of-concept substrate, minimal optimisation was required to show that judicious choice of catalyst loading and reaction temperature allowed control of the labelling pattern (Scheme 8). Specifically, labelling *ortho*- to the ketone group could be achieved with a 5 mol % catalyst loading of **1a** at room temperature to give **18**, whereas the globally-labelled product **19** could be obtained by employing 5 mol % of **1a** at 40 °C. In turn, the previously elusive nitro-selective product, **20**, was accessed by a retro-labelling strategy (employing H_2_ in place of D_2_) conducted on the globally-labelled product **19**.

## 3. Experimental Section

### 3.1. General Considerations

All reagents were obtained from commercial suppliers (Sigma-Aldrich, Gillingham, UK; Alfa Aesar, Heysham, UK; or Strem, Newton, UK) and used without further purification, unless otherwise stated. Dichloromethane was obtained from a PureSolv SPS-400-5 Solvent Purification System (Innovative Technology, Inc., Galway, Ireland), and deoxygenated by bubbling argon through for a minimum of ten minutes. Thin layer chromatography was carried out using Camlab silica plates coated with fluorescent indicator UV_254_ (Sigma-Aldrich), and were visualized using a Mineralight UVGL-25 lamp (Fisher Scientific UK Ltd., Loughborough, UK) or developed using vanillin solution. Catalysts **1a** [20], **1b** [16], and **1d** [18] were prepared according to literature procedures. Esters **6c** [32], **10a** [33], **10b** [34], **10c** [35], **10d** [36], **10e** [37], and **13** [38] were prepared according to the corresponding literature procedures. ^1^H-NMR spectra were recorded on a Bruker DPX 400 spectrometer (Bruker, Coventry, UK) at 400 MHz. Chemical shifts are reported in ppm and coupling constants are reported in Hz. All coupling constants are ^3^*J*_H–H_ unless otherwise stated.

### 3.2. General Procedures

#### 3.2.1. Deuteration of Substrates Using Iridium(I) Complexes **1a**, **1b**, **1d** and **5**

A three-necked round bottom flask was fitted with two stopcock side arms and a rubber septum, and then flame-dried. To this flask was added the iridium(I) complex and substrate. The solvent, DCM (2.5 mL, unless stated otherwise), was added, rinsing the inner walls of the flask, and the rubber septum was replaced with a greased glass stopper. The solution was placed under an atmosphere of Ar and stirred whilst being cooled to −78 °C in a dry ice/acetone bath. The flask was evacuated then refilled with argon and this process repeated. Upon a third evacuation, an atmosphere of deuterium gas was introduced to the flask. After sealing the flask, the cold bath was removed and the flask heated in an oil bath to the desired temperature. The reaction mixture was stirred for the allotted reaction time before removing the deuterium atmosphere and replacing with air. The resulting solution was washed with DCM and transferred to a single-necked flask before removing the solvent under reduced pressure. The catalyst was triturated from the remaining residue by addition of diethyl ether (3 × 5 mL). The solution was filtered through a short plug of silica before the solvent was removed *in vacuo* to deliver the crude product for analysis of the deuterium incorporation.

The level of deuterium incorporation in the substrate was determined by ^1^H-NMR spectroscopy. The integrals were calibrated against a peak corresponding to a position not expected to be labelled. Equation (1) was then used to calculate the extent of labelling:

(1) $$\%\;\text{Deuteration}=100-\left[\left(\frac{\text{residual integral}}{\text{number of labelling sites}}\right)\times \;100\right]$$

#### 3.2.2. Deuteration of Substrates for Rate Studies

A three-necked round bottom flask fitted with one stopcock side arm and two rubber septa was flame-dried. To this flask was added the iridium(I) complex, and substrate. The solvent, DCM (25 mL), was added, rinsing the inner walls of the flask, and one rubber septum was replaced with a greased glass stopper. The solution was placed under an atmosphere of argon and stirred whilst being cooled to −78 °C in a dry ice/acetone bath. The flask was evacuated then refilled with argon and this process repeated. Upon a third evacuation, an atmosphere of deuterium gas was introduced to the flask via a balloon. The balloon was left in place for the duration of the reaction to ensure a continuous supply of deuterium. The cold bath was removed and the flask heated in an oil bath to the desired temperature. The reaction mixture was then stirred for the allotted reaction time. An aliquot (1 mL) of the reaction mixture was removed at intervals throughout the reaction (10, 20, 30, 40, 50, 60 min, 2 h, and 18 h). Each aliquot was transferred to a vial containing diethyl ether. After removal of solvent *in vacuo*, the residue was analysed by ^1^H NMR spectroscopy. The integrals were calibrated against a peak corresponding to a position not expected to be labelled. The extent of labelling was determined using Equation (1).

### 3.3. Labelling Studies

#### 3.3.1. Deuteration of Ethyl benzoate **6a** with Complex **5**


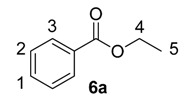


^1^H-NMR (400 MHz, CDCl_3_): δ 8.05 (d, *J* = 7.1 Hz, 2H, CH^3^), 7.54 (t, *J* = 7.3 Hz, 1H, CH^1^), 7.43 (t, *J* = 7.9 Hz, 2H, CH^2^), 4.38 (q, *J* = 7.2 Hz, 2H, CH_2_^4^), 1.39 (t, *J* = 7.1 Hz, 3H, CH_3_^5^). Incorporation expected at δ 8.05. Determined against integral at δ 1.39. Following *General Procedure A*, results are reported as (a) amount of substrate; (b) amount of catalyst; (c) reaction time; (d) reaction temperature; and (e) level of incorporation.

*Run 1*: (a) **6a**, 0.032 g, 0.215 mmol; (b) **5**, 8.04 mg, 0.01 mmol; (c) 1 h; (d) 25 °C; and (e) 17%.

*Run 2*: (a) **6a**, 0.032 g, 0.215 mmol; (b) **5**, 8.04 mg, 0.01 mmol; (c) 1 h; (d) 25 °C; and (e) 22%

#### 3.3.2. Deuteration of Ethyl 4-methylbenzoate **6b** with Complex **5**


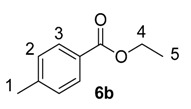


^1^H-NMR (400 MHz, CDCl_3_): δ 7.91 (d, *J* = 8.0 Hz, 2H, CH^3^), 7.21 (d, *J* = 7.9 Hz, 2H, CH^2^), 4.34 (q, *J* = 7.1 Hz, 2H, CH_2_^4^), 2.38 (s, 3H, CH_3_^1^) 1.36 (t, *J* = 7.1 Hz, 3H, CH_3_^5^). Incorporation expected at δ 7.91. Determined against integral at δ 2.38. Following *General Procedure A*, results are reported as (a) amount of substrate; (b) amount of catalyst; (c) reaction time; (d) reaction temperature; and (e) level of incorporation.

*Run 1*: (a) **6b**, 0.035 g, 0.215 mmol; (b) **5**, 8.04 mg, 0.01 mmol; (c) 1 h; (d) 25 °C; and (e) 36%.

*Run 2*: (a) **6b**, 0.035 g, 0.215 mmol; (b) **5**, 8.04 mg, 0.01 mmol; (c) 1 h; (d) 25 °C; and (e) 41%.

#### 3.3.3. Deuteration of Ethyl 4-(trifluoromethyl)benzoate **6c** with Complex **5**


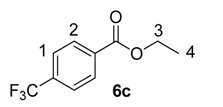


^1^H-NMR (400 MHz, CDCl_3_): δ 8.12 (d, *J =* 8.3 Hz, 2H, CH^2^) 7.67(d, *J =* 8.3 Hz, 2H, CH^1^) 4.38 (q, *J* = 7.2 Hz, 2H, CH_2_^3^), 1.38 (t, *J* = 7.2 Hz, 3H, CH_3_^4^). Incorporation expected at δ 8.12. Determined against integral at δ 1.38. Following *General Procedure A*, results are reported as (a) amount of substrate; (b) amount of catalyst; (c) reaction time; (d) reaction temperature; and (e) level of incorporation.

*Run 1*: (a) **6c**, 0.044 g, 0.215 mmol; (b) **5**, 8.04 mg, 0.01 mmol; (c) 1 h; (d) 25 °C; and (e) 8%

*Run 2*: (a) **6c**, 0.044 g, 0.215 mmol; (b) **5**, 8.04 mg, 0.01 mmol; (c) 1 h; (d) 25 °C; and (e) 11%

#### 3.3.4. Deuteration of Ethyl 4-chlorobenzoate **6d** with Complex **5**


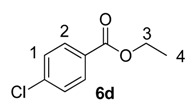


^1^H-NMR (400 MHz, CDCl_3_): δ 7.95 (d, *J =* 8.8 Hz, 2H, CH^2^) 7.38 (d, *J =* 8.8 Hz, 2H, CH^1^) 4.34 (q, *J* = 7.3 Hz, 2H, CH_2_^3^), 1.36 (t, *J* = 7.3 Hz, CH_3_^4^). Incorporation expected at δ 7.95. Determined against integral at δ 1.36. Following *General Procedure A*, results are reported as (a) amount of substrate; (b) amount of catalyst; (c) reaction time; (d) reaction temperature; and (e) level of incorporation.

*Run 1*: (a) **6d**, 0.039 g, 0.215 mmol; (b) **5**, 8.04 mg; 0.01 mmol; (c) 1 h; (d) 25 °C; and (e) 50%

*Run 2*: (a) **6d**, 0.039 g, 0.215 mmol; (b) **5**, 8.04 mg, 0.01 mmol; (c) 1 h; (d) 25 °C; and (e) 59%

#### 3.3.5. Deuteration of Ethyl 4-methoxybenzoate **6e** with Complex **5**


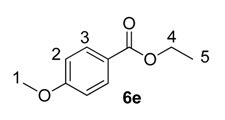


^1^H-NMR (400 MHz, CDCl_3_): δ 7.98 (d, *J* = 9.1 Hz, 2H, CH^3^), 6.89 (d, *J* = 9.0 Hz, 2H, CH^2^), 4.30 (q, *J =* 7.0 Hz, 2H, CH_2_^4^), 3.83 (s, 3H, CH_3_^1^), 1.35 (t, 7.1Hz, 3H, CH_3_^5^). Incorporation expected at δ 7.98. Determined against integral at δ 3.83. Following *General Procedure A*, results are reported as (a) amount of substrate; (b) amount of catalyst; (c) reaction time; (d) reaction temperature; and (e) level of incorporation.

*Run 1*: (a) **6e**, 0.038 g, 0.215 mmol; (b) **5**, 8.04 mg, 0.01 mmol; (c) 1 h; (d) 25 °C; and (e) 89%

*Run 2*: (a) **6e**, 0.038 g, 0.215 mmol; (b) **5**, 8.04 mg, 0.01 mmol; (c) 1 h; (d) 25 °C; and (e) 71%

#### 3.3.6. Deuteration of Esters **6a**–**e** Using Catalysts **1b** and **1a**

For the results relating to catalysts **1b** and **1a** in Scheme 2, please refer to the spectroscopic data from Section 3.3.1, Section 3.3.2, Section 3.3.3, Section 3.3.4 and Section 3.3.5 for the analysis of the deuterated esters **6a**–**e**. As catalyst type and amount used are the only variables changed, *General Procedure A* was followed with the results tabulated in Table 3.

#### 3.3.7. Deuteration of Methyl Benzoate **8a** with Complex **1a**


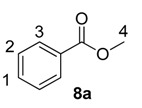


^1^H-NMR (400 MHz, CDCl_3_): δ 8.04 (d, *J =* 7.0 Hz, 2H, CH^3^), 7.56 (t, *J =* 7.4 Hz, 1H, CH^1^), 7.44 (t, *J* = 7.9 Hz, 2H, CH^2^), 3.92 (s, 3H, CH_3_^4^). Incorporation expected at δ 8.04. Determined against integral at δ 3.92. Following *General Procedure A*, results are reported as (a) amount of substrate; (b) amount of catalyst; (c) reaction time; (d) reaction temperature; and (e) level of incorporation.

*Run 1*: (a) **8a**, 0.032 g, 0.215 mmol; (b) **1a**, 10.1 mg, 0.01 mmol; (c) 1 h; (d) 25 °C; and (e) 52%

*Run 2*: (a) **8a**, 0.032 g, 0.215 mmol; (b) **1a**, 10.1 mg, 0.01 mmol; (c) 1 h; (d) 25 °C; and (e) 51%

**Table 3:** Deuteration of Esters **6a**–**e**.

Entry	Substrate	Catalyst	%D (run 1)	%D (run 2)
1	**6a**	**1b** (10.5 mg)	10	10
2	**6b**		15	23
3	**6c**		62	50
4	**6d**		85	93
5	**6e**		65	59
6	**6a**	**1a** (10.1 mg)	6	40
7	**6b**		53	83
8	**6c**		74	79
9	**6d**		95	95
10	**6e**		96	96

#### 3.3.8. Deuteration of Methyl 4-methylbenzoate **8b** with Complex **1a**


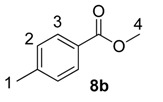


^1^H-NMR (400 MHz, CDCl_3_): δ 7.99 (d, *J =* 8.0 Hz, 2H, CH^3^), 7.21 (d, *J =* 8.0 Hz, 2H, CH^2^), 3.89 (s, 3H, CH_3_^4^), 2.39 (s, 3H, CH_3_^1^). Incorporation expected at δ 7.99. Determined against integral at δ 2.39. Following *General Procedure A*, results are reported as (a) amount of substrate; (b) amount of catalyst; (c) reaction time; (d) reaction temperature; and (e) level of incorporation.

*Run 1*: (a) **8b**, 0.035 g, 0.215 mmol; (b) **1a**, 10.1 mg, 0.01 mmol; (c) 1 h, d) 25 °C; and (e) 35%

*Run 2*: (a) **8b**, 0.035 g, 0.215 mmol; (b) **1a**, 10.1 mg, 0.01 mmol; (c) 1 h; (d) 25 °C; and (e) 48%

#### 3.3.9. Deuteration of Methyl 4-(trifluoromethyl)benzoate **8c** with Complex **1a**


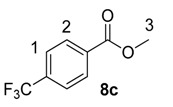


^1^H-NMR (400 MHz, CDCl_3_): δ 8.14 (d, *J =* 8.8 Hz, 2H, CH^2^), 7.70 (d, *J =* 8.8 Hz, 2H, CH^1^), 3.94 (s, 3H, CH_3_^3^). Incorporation expected at δ 8.14. Determined against integral at δ 3.94. Following *General Procedure A*, results are reported as (a) amount of substrate; (b) amount of catalyst; (c) reaction time; (d) reaction temperature; and (e) level of incorporation.

*Run 1*: (a) **8c**, 0.044 g, 0.215 mmol; (b) **1a**, 10.1 mg, 0.01 mmol; (c) 1 h; (d) 25 °C; and (e) 27%

*Run 2*: (a) **8c**, 0.044 g, 0.215 mmol; (b) **1a**, 10.1 mg, 0.01 mmol; (c) 1 h; (d) 25 °C; and (e) 36%

#### 3.3.10. Deuteration of Methyl 4-chlorobenzoate **8d** with Complex **1a**


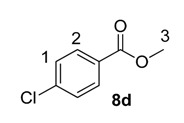


^1^H-NMR (400 MHz, CDCl_3_): δ 7.93 (d, *J =* 8.9 Hz, 2H, CH^2^), 7.36 (d, *J =* 8.9 Hz, 2H, CH^1^), 3.90 (s, 3H, CH_3_^3^). Incorporation expected at δ 7.93. Determined against integral at δ 3.90. Following *General Procedure A*, results are reported as (a) amount of substrate; (b) amount of catalyst; (c) reaction time; (d) reaction temperature; and (e) level of incorporation.

*Run 1*: (a) **8d**, 0.039 g, 0.215 mmol; (b) **1a**, 10.1 mg, 0.01 mmol; (c) 1 h; (d) 25 °C; and (e) 91%

*Run 2*: (a) **8d**, 0.039 g, 0.215 mmol; (b) **1a**, 10.1 mg, 0.01 mmol; (c) 1 h; (d) 25 °C; and (e) 94%

#### 3.3.11. Deuteration of Methyl 4-methoxybenzoate **8e** with Complex **1a**


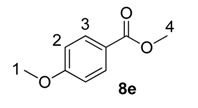


^1^H-NMR (400 MHz, CDCl_3_): δ 7.96 (d, *J =* 9.0 Hz, 2H, CH^3^), 6.89 (d, *J =* 9.0 Hz, 2H, CH^2^), 3.85 (s, 3H, CH_3_^4^), 3.82 (s, 3H, CH_3_^1^). Incorporation expected at δ 7.96. Determined against integral at δ 3.82. Following *General Procedure A*, results are reported as (a) amount of substrate; (b) amount of catalyst; (c) reaction time; (d) reaction temperature; and (e) level of incorporation.

*Run 1*: (a) **8e**, 0.038 g, 0.215 mmol; (b) **1a**, 10.1 mg, 0.01 mmol; (c) 1 h; (d) 25 °C; and (e) 87%

*Run 2*: (a) **8e**, 0.038 g, 0.215 mmol; (b) **1a**, 10.1 mg, 0.01 mmol; (c) 1 h; (d) 25 °C; and (e) 90%

#### 3.3.12. Deuteration of *n*-Propyl 4-methoxybenzoate **10a** with Complex **1a**


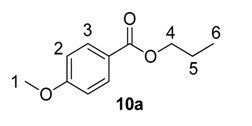


^1^H-NMR (400 MHz, CDCl_3_): δ 8.00 (d, *J* = 9.2 Hz, 2H, CH^3^), 6.92 (d, *J* = 9.2 Hz, 2H, CH^2^), 4.25 (t, *J* = 6.8 Hz, 2H, CH^4^), 3.86 (s, 3H, CH_3_^1^), 1.82–1.73 (m, 2H, CH_2_^5^), 1.02 (t, *J* = 7.3 Hz, 3H, CH_3_^6^). Incorporation expected at δ 8.00. Determined against integral at δ 3.86. Following *General Procedure A*, results are reported as (a) amount of substrate; (b) amount of catalyst; (c) reaction time; (d) reaction temperature; and (e) level of incorporation.

*Run 1*: (a) **10a**, 0.042 g, 0.215 mmol; (b) **1a**, 10.9 mg, 0.01 mmol; (c) 1 h; (d) 25 °C; and (e) 28%

*Run 2*: (a) **10a**, 0.038 g, 0.215 mmol; (b) **1a**, 10.9 mg, 0.01 mmol; (c) 1 h; (d) 25 °C; and (e) 27%

#### 3.3.13. Deuteration of 2,2,2-Trifluoroethyl 4-methoxybenzoate **10b** with Complex **1a**


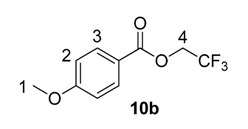


^1^H-NMR (400 MHz, CDCl_3_): δ 8.06 (d, *J* = 9.3 Hz, 2H, CH^3^), 6.97 (d, *J* = 9.3 Hz, 2H, CH^2^), 4.69 (q, ^3^*J*_H-F_ = 8.5 Hz, 2H, CH_2_^4^), 3.90 (s, 3H, CH_3_^1^). Incorporation expected at δ 8.06. Determined against integral at δ 3.90. Following *General Procedure A*, results are reported as (a) amount of substrate; (b) amount of catalyst; (c) reaction time; (d) reaction temperature; and (e) level of incorporation.

*Run 1*: (a) **10b**, 0.050 g, 0.215 mmol; (b) **1a**, 10.9 mg, 0.01 mmol; (c) 1 h; (d) 25 °C; and (e) 9%

*Run 2*: (a) **10b**, 0.050 g, 0.215 mmol; (b) **1a**, 10.9 mg, 0.01 mmol; (c) 1 h; (d) 25 °C; and (e) 7%

#### 3.3.14. Deuteration of *t*-Butyl 4-methoxybenzoate **10c** with Complex **1a**


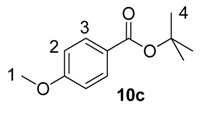


^1^H-NMR (400 MHz, CDCl_3_): δ 7.94 (d, *J* = 8.7 Hz, 2H, CH^3^), 6.89 (d, *J* = 8.9 Hz, 2H, CH^2^), 3.85 (s, 3H, CH_3_^1^), 1.58 (s, 9H, CH_3_^4^). Incorporation expected at δ 7.94. Determined against integral at δ 3.85. Following *General Procedure A*, results are reported as (a) amount of substrate, (b) amount of catalyst, (c) reaction time, (d) reaction temperature, and (e) level of incorporation.

*Run 1*: (a) **10c**, 0.045 g, 0.215 mmol; (b) **1a**, 10.9 mg, 0.01 mmol; (c) 1 h; (d) 25 °C; and (e) 10%

*Run 2*: (a) **10c**, 0.045 g, 0.215 mmol; (b) **1a**, 10.9 mg, 0.01 mmol; (c) 1 h; (d) 25 °C; and (e) 9%

#### 3.3.15. Deuteration of Iso-propyl 4-methoxybenzoate **10d** with Complex **1a**


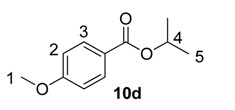


^1^H-NMR (400 MHz, CDCl_3_): δ 7.97 (d, *J* = 9.0 Hz, 2H, CH^3^), 6.89 (d, *J* = 9.0 Hz, 2H, CH^2^), 5.20 (septet, *J* = 6.3 Hz, 1H, CH^4^), 3.84 (s, 3H, CH_3_^1^), 1.33 (d, *J* = 6.5 Hz, 6H, CH_3_^5^). Incorporation expected at δ 7.97. Determined against integral at δ 3.84. Following *General Procedure A*, results are reported as (a) amount of substrate; (b) amount of catalyst; (c) reaction time; (d) reaction temperature; and (e) level of incorporation.

*Run 1*: (a) **10d**, 0.042 g, 0.215 mmol; (b) **1a**, 10.9 mg, 0.01 mmol; (c) 1 h; (d) 25 °C; and (e) 79%

*Run 2*: (a) **10d**, 0.042 g, 0.215 mmol; (b) **1a**, 10.9 mg, 0.01 mmol; (c) 1 h; (d) 25 °C; and (e) 66%

#### 3.3.16. Deuteration of Benzyl 4-methoxybenzoate **10e** with Complex **1a**


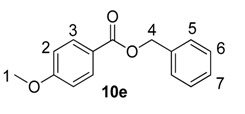


^1^H-NMR (400 MHz, CDCl_3_): δ 8.04 (d, *J* = 9.0 Hz, 2H, CH^3^), 7.45–7.43 (m, 2H, CH^5^), 7.40–7.37 (m, 2H, CH^6^), 7.35–7.32 (m, 1H, CH^7^), 6.92 (d, *J* = 9.0 Hz, 2H, CH^2^), 5.34 (s, 2H, CH_2_^4^), 3.90 (s, 3H, CH_3_^1^). Incorporation expected at δ 8.04. Determined against integral at δ 3.90. Following *General Procedure A*, results are reported as (a) amount of substrate; (b) amount of catalyst; (c) reaction time; (d) reaction temperature; and (e) level of incorporation.

*Run 1*: (a) **10e**, 0.052 g, 0.215 mmol; (b) **1a**, 10.9 mg, 0.01 mmol; (c) 1 h; (d) 25 °C; and (e) 73%

*Run 2*: (a) **10e**, 0.052 g, 0.215 mmol; (b) **1a**, 10.9 mg, 0.01 mmol; (c) 1 h; (d) 25 °C; and (e) 51%

### 3.4. Temperature Effects

For the results relating to Scheme 3, readers are directed to the spectroscopic data in the relevant parts of Section 3.3 for the analysis of the corresponding deuterated esters **6a**–**c** and **8a**–**c**. As catalyst type and amount used are the only variables changed, the remaining results are tabulated below. In all cases, 0.215 mmol of substrate was employed with 10.1 mg of catalyst **1a** (0.01 mmol, 5 mol %) and the reactions run at 40 °C, otherwise following *General Procedure A*, and the results shown below in Table 4.

**Table 4:** Deuteration of Esters **6a**–**c** and **8a**–**c** at 40 °C.

Entry	Substrate	%D (run 1)	%D (run 2)
1	**6a**	85	86
2	**6b**	89	91
3	**6c**	95	96
4	**8a**	75	76
5	**8b**	96	95
6	**8c**	92	93

### 3.5. Catalyst Counterion Effects

#### 3.5.1. Deuteration of *n*-Propyl 4-methoxybenzoate **10a** with Complex **1d**

Following *General Procedure A*, results are reported as (a) amount of substrate; (b) amount of catalyst; (c) reaction time; (d) reaction temperature; and (e) level of incorporation. 

(a) **10a**, 0.042 g, 0.215 mmol; (b) **1d**, 18.6 mg, 0.01 mmol; (c) 1 h; (d) 25 °C; and (e) 89%

#### 3.5.2. Deuteration of 2,2,2-Trifluoroethyl 4-methoxybenzoate **10b** with Complex **1d**

Following *General Procedure A*, results are reported as (a) amount of substrate; (b) amount of catalyst; (c) reaction time; (d) reaction temperature; and (e) level of incorporation.

(a) **10b**, 0.050 g, 0.215 mmol; (b) **1d**, 18.6 mg, 0.01 mmol; (c) 1 h; (d) 25 °C; and (e) 24%

#### 3.5.3. Deuteration of *t*-Butyl 4-methoxybenzoate **10c** with Complex **1d**

Following *General Procedure A*, results are reported as (a) amount of substrate; (b) amount of catalyst; (c) reaction time; (d) reaction temperature; and (e) level of incorporation.

(a) **10c**, 0.045 g, 0.215 mmol; (b) **1d**, 18.6 mg, 0.01 mmol; (c) 1 h, (d) 25 °C; and (e) 41%

#### 3.5.4. Deuteration of *iso*-Propyl 4-methoxybenzoate **10d** with Complex **1d**

Following *General Procedure A*, results are reported as (a) amount of substrate; (b) amount of catalyst; (c) reaction time; (d) reaction temperature; and (e) level of incorporation.

(a) **10d**, 0.042 g 0.215 mmol; (b) **1d**, 18.6 mg, 0.01 mmol; (c) 1 h; (d) 25 °C; and (e) 94%.

#### 3.5.5. Deuteration of Benzyl 4-methoxybenzoate **10e** with Complex **1d**

Following *General Procedure A*, results are reported as (a) amount of substrate; (b) amount of catalyst; (c) reaction time; (d) reaction temperature; and (e) level of incorporation.

(a) **10e**, 0.052 g, 0.215 mmol; (b) **1d**, 18.6 mg, 0.01 mmol; (c) 1 h; (d) 25 °C; and (e) 89%.

### 3.6. Chemoselectivity Studies

#### 3.6.1. Deuteration of Ethyl 4-nitrobenzoate **12** with Complex **1a**


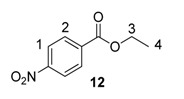


^1^H-NMR (400 MHz, CDCl_3_): δ 8.27 (d, *J =* 9.0 Hz, 2H, CH^1^), 8.19 (d, *J =* 8.3 Hz, 2H, CH^2^), 4.42 (q, *J* = 7.5 Hz, 2H, CH_2_^3^), 1.41 (t, *J* = 7.5 Hz, 3H, CH_3_^4^). Incorporation expected at δ 8.27 and 8.19. Determined against integral at δ 1.41. Following *General Procedure A*, results are reported as (a) amount of substrate; (b) amount of catalyst; (c) reaction time; (d) reaction temperature; and (e) level of incorporation at position 1 and position 2.

*Run 1*: (a) **12**, 0.042 g, 0.215 mmol; (b) **1a**, 10.1 mg, 0.01 mmol; (c) 1 h; (d) 25 °C; and (e) 1: 72%, 2: 49%

*Run 2*: (a) **12**, 0.042 g, 0.215 mmol; (b) **1a**, 10.1 mg, 0.01 mmol; (c) 1 h; (d) 25 °C; and (e) 1: 72%, 2: 52%

#### 3.6.2. Deuteration of *N*,*N*-Diethyl 4-nitrobenzamide **13** with Complex **1a**


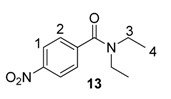


^1^H-NMR (400 MHz, CDCl_3_): δ 8.28 (d, *J* = 8.9 Hz, 2H, CH^1^), 7.57 (d, *J =* 8.9 Hz, 2H, CH^2^), 3.17–3.53 (m, 4H, CH_2_^3^), 1.25 (br s, 6H, CH_3_^4^). Incorporation expected at δ 8.28 and 7.57. Determined against integral at δ 1.25. Following *General Procedure A*, results are reported as (a) amount of substrate; (b) amount of catalyst; (c) reaction time; (d) reaction temperature; and (e) level of incorporation at position 1 and position 2.

*Run 1*: (a) **13**, 0.047 g, 0.215 mmol; (b) **1a**, 10.1 mg, 0.01 mmol; (c) 1 h; (d) 25 °C; and (e) 1: 89%, 2: 93%.

*Run 2*: (a) **13**, 0.047 g, 0.215 mmol; (b) **1a**, 10.1 mg, 0.01 mmol; (c) 1 h, d) 25 °C, and (e) 1: 94%, 2: 93%.

#### 3.6.3. Deuteration of 4-Nitroacetophenone **14** with Complex **1a**


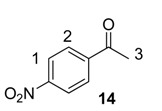


^1^H-NMR (400 MHz, CDCl_3_): δ 8.32 (d, *J* = 8.9 Hz, 2H, CH^1^), 8.11 (d, *J =* 8.9 Hz, 2H, CH^2^), 2.69 (s, 1H, CH_3_^3^). Incorporation expected at δ 8.32 and 8.11. Determined against integral at δ 2.69. Following *General Procedure A*, results are reported as (a) amount of substrate; (b) amount of catalyst; (c) reaction time; (d) reaction temperature; and (e) level of incorporation at position 1 and position 2.

*Run 1*: (a) **14**, 0.0467 g, 0.215 mmol; (b) **1a**, 10.1 mg, 0.01 mmol; (c) 1 h; (d) 25 °C; and (e) 1: 28%, 2: 97%

*Run 2*: (a) **14**, 0.0467 g, 0.215 mmol; (b) **1a**, 10.1 mg, 0.01 mmol; (c) 1 h; (d) 25 °C; and (e) 1: 33%, 2: 85%

### 3.7. Rate Studies

#### 3.7.1. Deuteration of Ethyl 4-nitrobenzoate **12** with Complex **1a**


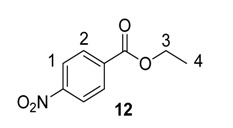


^1^H-NMR (400 MHz, CDCl_3_): δ 8.27 (d, *J =* 9.0 Hz, 2H, CH^1^), 8.19 (d, *J =* 8.3 Hz, 2H, CH^2^). 4.42 (q, *J* = 7.5 Hz, 2H, CH_2_^3^), 1.41 (t, *J* = 7.5 Hz, 3H, CH_3_^4^). Incorporation expected at 1: δ 8.27 and 2: δ 8.19. Determined against integral at δ 1.41. Following *General Procedure B*, results are reported as (a) amount of substrate; (b) amount of catalyst; (c) reaction time; (d) reaction temperature; and (e) level of incorporation at position 1 and 2 at each time interval.

*Labelling at Position 1*: (a) **12**, 0.399 g, 2.15 mmol; (b) **1a**, 2.17 mg, 0.0215 mmol; (c) 18 h; (d) 25 °C; and (e) 21% (10 min), 37% (20 min), 47% (30 min), 56% (40 min), 61% (50 min), 64% (1 h), 73% (2 h), and 76% (18 h).

*Labelling at Position 2*: (a) **12**, 0.399 g, 2.15 mmol; (b) **1a**, 2.17 mg, 0.0215 mmol; (c) 18 h; (d) 25 °C; and (e) 2: 14% (10 min), 24% (20 min), 33% (30 min), 37% (40 min), 41% (50 min), 43% (1 h), 52% (2 h), and 56% (18 h).

#### 3.7.2. Deuteration of 4-Nitroacetophenone **14** with Complex **1a**


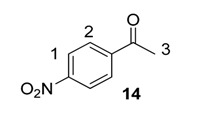


^1^H-NMR (400 MHz, CDCl_3_): δ 8.32 (d, *J* = 8.9 Hz, 2H, CH^1^), 8.11 (d, *J =* 8.9 Hz, 2H, CH^2^), 2.69 (s, 1H, CH_3_^3^). Incorporation expected at δ 8.32 and 8.11. Determined against integral at δ 2.69. Following *General Procedure B*, results are reported as (a) amount of substrate; (b) amount of catalyst; (c) reaction time; (d) reaction temperature; and (e) level of incorporation at position 1 and 2 at each time interval.

*Labelling at Position 1*: (a) **14**, 0.355 g, 2.15 mmol; (b) **1a**, 2.12 mg, 0.021 mmol; (c) 18 h; (d) 25 °C; and (e) 8% (10 min), 13% (20 min), 19% (30 min), 26%(40 min), 29% (50 min), 33% (1 h), 65% (2 h), 69% (18 h).

*Labelling at Position 2*: (a) **14**, 0.0467 g, 2.15 mmol; (b) **1a**, 2.12 mg, 0.01 mmol; (c) 18 h; (d) 25 °C; and (e) 54% (10 min), 75% (20 min), 82% (30 min), 84% (40 min), 85% (50 min), 85% (1 h), 91% (2 h), 95% (18 h).

### 3.8. Practical Exploitation of Directing Group Chemoselectivity


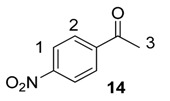


#### 3.8.1. Deuteration of 4-Nitroacetophenone **14** with Complex **1a**

^1^H-NMR (400 MHz, CDCl_3_): δ 8.32 (d, *J* = 8.9 Hz, 2H, CH^1^), 8.11 (d, *J =* 8.9 Hz, 2H, CH^2^), 2.69 (s, 1H, CH_3_^3^). Incorporation expected at δ 8.32 and 8.11. Determined against integral at δ 2.69. See the details within Section 3.6.3.

#### 3.8.2. Deuteration of 4-Nitroacetophenone **14** with Complex **1a**

Following *General Procedure A*, results are reported as (a) amount of substrate; (b) amount of catalyst; (c) reaction time, (d) reaction temperature, and (e) level of incorporation at position 1 and position 2.

(a) **14**, 0.035 g, 0.215 mmol; (b) **1a**, 10.1 mg, 0.01 mmol; (c) 1 h; (d) 40 °C; and (e) 1: 97%, 2: 97%

#### 3.8.3. Hydrogenation of 4-Nitroacetophenone-*d_4_* **19** with Complex **1a**

Following *General Procedure A* (deuterium gas was replaced with hydrogen gas), results are reported as (a) amount of substrate; (b) amount of catalyst; (c) reaction time; (d) reaction temperature; and (e) level of incorporation of deuterium at position 1 and position 2.

(a) **19**, 0.0363 g, 0.215 mmol; (b) **1a**, 2.1 mg, 0.0021 mmol; (c) 1 h; (d) 25 °C; and (e) 1: 90%, 2: 12%.

## 4. Computational Details

DFT [39] was employed to calculate the gas-phase electronic structures and energies for all species involved in H/D exchange reactions. All structures have been optimised with the hybrid meta-GGA exchange correlation functional M06 [40]. The M06 density functional was used in conjunction with the 6-31G(d) basis set for main group non-metal atoms and the Stuttgart RSC [41] effective core potential along with the associated basis set for Ir. The participating transition states (TS) are located at the same level of theory. Harmonic vibrational frequencies are calculated at the same level of theory to characterize respective minima (reactants, intermediates, and products with no imaginary frequency) and first order saddle points (TSs with one imaginary frequency). The validity of using the 6-31G(d) basis set has previously been checked by comparative single point energy calculations employing the def2-TZVP basis set for all atoms on similar H/D exchange systems [20]. All calculations using the M06 functional have been performed using Gaussian 09 quantum chemistry program package (version A.02). Calculations were first carried out in the gas phase before reoptimising each structure at the same level of theory, implementing the Polarizable Continuum Model (PCM) for DCM as the solvent [42]. All coordinates provided are listed in Cartesian format, with charge and multiplicity of each system given at the top of the coordinate list (*i.e*., 0 1 = neutral singlet; 1 1 = 1 + charged singlet).

## 5. Conclusions 

In summary, we have divulged novel iridium-catalysed methods for the *ortho*-deuteration of benzoate esters by the application of complexes emerging from our laboratory, possessing a bulky NHC/phosphine combination. Inherent variability in reproducibly labelling ester substrates to useable levels of d-incorporation was solved by two methods: (i) a mild increase in reaction temperature; and (ii) a switch in the catalyst anion from PF_6_ to BArF; this delivered good to excellent levels of deuterium incorporation adjacent to ester directing groups. Kinetic studies on intramolecular directing group chemoselectivity revealed that selectivity *vs.* time is substrate dependent, showing the possibility that different levels of binding conformer equilibria are possible. Supporting DFT analyses of the systems studied experimentally support previous findings that suggested the most stable binding conformer is also the most reactive. We have demonstrated that such knowledge can be exploited experimentally and, as such, we have shown that different modes of regioselective labelling is possible in a multifunctional substrate by simple variation of the reaction conditions. Overall, we believe that these methods further enhance the applicable substrate scope and wider utility of the iridium complexes at the centre of this study.

## Supplementary Materials

Supplementary materials can be accessed at: http://www.mdpi.com/1420-3049/20/07/11676/s1.

## Notes

*Sample Availability: *Not available.

## References

[1] Isin E.M., Elmore C.S., Nilsson G.N., Thompson R.A., Weidolf L. (2012). Use of radiolabeled compounds in drug metabolism and pharmacokinetic studies. Chem. Res. Toxicol..

[2] Allen P.H., Hickey M.J., Kingston L.P., Wilkinson D.J. (2010). Metal-catalysed isotopic exchange labelling: 30 years of experience in pharmaceutical R&D. J. Label. Compd. Radiopharm..

[3] Hesk D., Lavey C.F., McNamara P. (2010). Tritium labelling of pharmaceuticals by metal-catalysed exchange methods. J. Label. Compd. Radiopharm..

[4] Heys J.R. (2007). Organoiridium complexes for hydrogen isotope exchange labeling. J. Label. Compd. Radiopharm..

[5] Lockley W.J.S., McEwen A., Cooke R. (2012). Tritium: A coming of age for drug discovery and development ADME studies. J. Label. Compd. Radiopharm..

[6] Nilsson G.N., Kerr W.J. (2010). The development and use of novel iridium complexes as catalysts for ortho-directed hydrogen isotope exchange reactions. J. Label. Compd. Radiopharm..

[7] Sawama Y., Monguchi Y., Sajiki H. (2012). Efficient H-D Exchange Reactions Using Heterogeneous Platinum-Group Metal on Carbon-H_2_-D_2_O System. Synlett.

[8] Atzrodt J., Derdau V., Fey T., Zimmermann J. (2007). The renaissance of H/D exchange. Angew. Chem. Int. Ed..

[9] Lockley W.J.S. (2007). 30 Years with ortho-directed hydrogen isotope exchange labelling. J. Label. Compd. Radiopharm..

[10] Hesk D., McNamara P. (2007). Synthesis of isotopically labelled compounds at Schering-Plough, an historical perspective. J. Label. Compd. Radiopharm..

[11] Lin D., Chang W. (2000). Chemical derivatization and the selection of deuterated internal standard for quantitative determination—Methamphetamine example. J. Anal. Toxicol..

[12] Atzrodt J., Derdau V. (2010). Pd- and Pt-catalyzed H/D exchange methods and their application for internal MS standard preparation from a Sanofi-Aventis perspective. J. Label. Compd. Radiopharm..

[13] Parkin G. (2007). Applications of deuterium isotope effects for probing aspects of reactions involving oxidative addition and reductive elimination of H–H and C–H bonds. J. Label. Compd. Radiopharm..

[14] Heinekey D.M. (2007). Transition metal dihydrogen complexes: Isotope effects on reactivity and structure. J. Label. Compd. Radiopharm..

[15] Quasdorf K.W., Huters A.D., Lodewyk M.W., Tantillo D.J., Garg N.K. (2012). Total synthesis of oxidized welwitindolinones and (−)-*N*-methylwelwitindolinone C isonitrile. J. Am. Chem. Soc..

[16] Brown J.A., Irvine S., Kennedy A.R., Kerr W.J., Andersson S., Nilsson G.N. (2008). Highly active iridium(I) complexes for catalytic hydrogen isotope exchange. Chem. Commun..

[17] Cochrane A.R., Idziak C., Kerr W.J., Mondal B., Paterson L.C., Tuttle T., Andersson S., Nilsson G.N. (2014). Practically convenient and industrially-aligned methods for iridium-catalysed hydrogen isotope exchange processes. Org. Biomol. Chem..

[18] Kennedy A.R., Kerr W.J., Moir R., Reid M. (2014). Anion effects to deliver enhanced iridium catalysts for hydrogen isotope exchange processes. Org. Biomol. Chem..

[19] Atzrodt J., Derdau V., Kerr W.J., Reid M., Rojahn P., Weck R. (2015). Expanded applicability of iridium(I) NHC/phosphine catalysts in hydrogen isotope exchange processes with pharmaceutically-relevant heterocycles. Tetrahedron.

[20] Kerr W.J., Reid M., Tuttle T. (2015). Iridium-Catalyzed C–H Activation and Deuteration of Primary Sulfonamides: An Experimental and Computational Study. ACS Catal..

[21] Cochrane A.R., Irvine S., Kerr W.J., Reid M., Andersson S., Nilsson G.N. (2013). Application of Neutral Iridium(I) *N*-Heterocyclic Carbene Complexes in *ortho*-Directed Hydrogen Isotope Exchange. J. Label. Compd. Radiopharm..

[22] Kerr W.J., Mudd R.J., Paterson L.C., Brown J.A. (2014). Iridium(I)-Catalyzed Regioselective C–H Activation and Hydrogen-Isotope Exchange of Non-aromatic Unsaturated Functionality. Chem. Eur. J..

[23] Cross P.W.C., Ellames G.J., Gibson J.S., Herbert J.M., Kerr W.J., McNeill A.H., Mathers T.W. (2003). Conditions for deuterium exchange mediated by iridium complexes formed *in situ*. Tetrahedron.

[24] Ellames G., Gibson J., Herbert J., McNeill A. (2001). The scope and limitations of deuteration mediated by Crabtree’s catalyst. Tetrahedron.

[25] Heys J.R., Shu A.Y.L., Senderoff S.G., Phillips N.M. (1993). Deuterium exchange labelling of substituted aromatics using [IrH_2_(Me_2_CO)_2_(PPh_3_)_2_BF_4_. J. Label. Compd. Radiopharm..

[26] Shu A., Chen W., Heys J. (1996). Organoiridium catalyzed hydrogen isotope exchange: ligand effects on catalyst activity and regioselectivity. J. Organomet. Chem..

[27] Piola L., Fernández-Salas J.A., Manzini S., Nolan S.P. (2014). Regioselective ruthenium catalysed H–D exchange using D_2_O as the deuterium source. Org. Biomol. Chem..

[28] Hesk D., Das P.R., Evans B. (1995). Deuteration of Acetanilides and Other Substituted Aromatics Using [Ir(COD)(Cy_3_P)Py)PF_6_ as Catalyst. J. Label. Compd. Radiopharm..

[29] Brown J.A., Cochrane A.R., Irvine S., Kerr W.J., Mondal B., Parkinson J.A., Paterson L.C., Reid M., Tuttle T., Andersson S. (2014). The Synthesis of Highly Active Iridium(I) Complexes and their Application in Catalytic Hydrogen Isotope Exchange. Adv. Synth. Catal..

[30] Hansch C., Leo A., Taft R.W. (1991). A survey of Hammett substituent constants and resonance and field parameters. Chem. Rev..

[31] Seeman J.I. (1983). Effect of conformational change on reactivity in organic chemistry. Evaluations, applications, and extensions of Curtin-Hammett Winstein-Holness kinetics. Chem. Rev..

[32] Shang R., Fu Y., Li J.B., Zhang S.L., Guo Q.X., Liu L. (2009). Synthesis of Aromatic Esters via Pd-Catalyzed Decarboxylative Coupling of Potassium Oxalate Monoesters with Aryl Bromides and Chlorides. J. Am. Chem. Soc..

[33] Moller H., Thimm H.J. Cosmetic Preparations with Alkoxybenzoic Acid Esters as Inflammation Inhibitors and Method.

[34] Mori N., Togo H. (2005). Facile oxidative conversion of alcohols to esters usingmolecular iodine. Tetrahedron.

[35] Jackson R.W. (2001). A mild and selective method for the cleavage of *tert*-butyl esters. Tetrahedron Lett..

[36] Kuh L.P. (1949). Ionization of Organic Esters in Sulfuric Acid. II. Alkyl Oxygen Fission. J. Am. Chem. Soc..

[37] Rivero-Cruz B., Rivero-Cruz I., Rodríguez-Sotres R., Mata R. (2007). Effect of natural and synthetic benzyl benzoates on calmodulin. Phytochemistry.

[38] Kaur G., Narayanan V.L., Risbood P.A., Hollingshead M.G., Stinson S.F., Varma R.K., Sausville E.A. (2005). Synthesis, structure–activity relationship, and p210^bcr−abl^ protein tyrosine kinase activity of novel AG 957 analogs. Bioorg. Med. Chem..

[39] Kohn W., Sham L. (1965). Self-consistent equations including exchange and correlation effects. Phys. Rev..

[40] Zhao Y., Truhlar D.G. (2008). The M06 suite of density functionals for main group thermochemistry, thermochemical kinetics, noncovalent interactions, excited states, and transition elements: two new functionals and systematic testing of four M06-class functionals and 12 other function. Theor. Chem. Acc..

[41] Andrae D., Häußerman U., Dolg M., Stoll H., Preuß H. (1990). Energy-adjusted ab initio pseudopotentials for the second and third row transition elements. Theor. Chim. Acta.

[42] Tomasi J., Mennucci B., Cammi R. (2005). Quantum Mechanical Continuum Solvation Models. Chem. Rev..

