# Supplementary material for: Iridium-Catalysed ortho-Directed Deuterium Labelling of Aromatic Esters—An Experimental and Theoretical Study on Directing Group Chemoselectivity
Source: Molecules. 2015 Jun 25;20(7):11676–98. doi: 10.3390/molecules200711676 (PMC6332247; doi:10.3390/molecules200711676)
Supplement: Supplementary file 1 [file molecules-20-11676-s001.pdf]

Supplementary Materials

Structural Coordinates of Binding Conformers (Gas Phase)—from Scheme 7

Substrate 12—Position 15 (nitro Bound Reactant)

1 1

C	2.16765800	4.40849900	−0.75910500
C	3.35316300	3.76210000	−0.80718400
N	1.20097100	3.44246900	−0.51859600
N	3.08613700	2.41740300	−0.59687100
C	4.11151500	1.41332600	−0.52887900
C	4.65065100	1.09391600	0.72403200
C	4.51642200	0.78393500	−1.70818500
C	5.61891700	0.09560900	0.77096900
C	5.49110800	−0.20914300	−1.60647500
C	6.04935900	−0.56789600	−0.38031800
H	6.05061900	−0.17493700	1.73619000
H	5.82360700	−0.71535900	−2.51476200
C	−0.19500600	3.72541500	−0.36258700
C	−0.66469800	4.08462700	0.90740700
C	−1.03921400	3.58950000	−1.46932200
C	−2.04027800	4.24795100	1.06215900
C	−2.40497000	3.78432800	−1.26524100
C	−2.92435900	4.09201400	−0.00740600
H	−2.43257200	4.49540100	2.05063500
H	−3.08619500	3.66562900	−2.11006100
C	−0.50622900	3.17973000	−2.80958500
H	0.36584600	3.77681100	−3.10824300
H	−0.17697900	2.12771500	−2.80665100
H	−1.27218300	3.28838300	−3.58536700
C	0.27448700	4.26121800	2.06215400
H	0.91953500	3.38176500	2.20084500
H	0.93794000	5.12410100	1.91178200
H	−0.27737900	4.42288500	2.99464000
C	−4.40123200	4.25037900	0.19141100
H	−4.71177700	3.90484000	1.18559300
H	−4.70478800	5.30298600	0.10679300
H	−4.96861700	3.68734700	−0.55998800
C	7.09242300	−1.63970500	−0.28368400
H	6.78171700	−2.42945300	0.41480000
H	7.28770800	−2.10505700	−1.25746300
H	8.04416700	−1.23913900	0.09005400
C	3.90449600	1.13958100	−3.02881000

H	2.83126000	0.89572500	−3.04821100
H	3.98910100	2.21258900	−3.24885900
H	4.38826100	0.59263100	−3.84540100
C	4.17318600	1.77306000	1.97161100
H	4.17035600	2.86751800	1.87340700
H	3.14378800	1.47334200	2.22001900
H	4.80812400	1.51327900	2.82560600
H	4.36313200	4.11669400	−0.96680500
H	1.90703600	5.45296800	−0.87027700
C	1.75364700	2.20118500	−0.41249600
Ir	0.87719400	0.34545500	−0.09338900
H	2.34297400	−0.10723500	0.12286000
H	1.29984300	−0.00684600	−1.56666800
P	0.34125500	−1.94654700	0.11301500
C	1.83806200	−2.97290900	0.34890800
C	2.91143900	−2.77093000	−0.52676600
C	1.93112900	−3.96163400	1.33090500
C	4.05991100	−3.54364500	−0.41673000
H	2.84812900	−2.00369100	−1.30053600
C	3.08875500	−4.72755100	1.44385500
H	1.10062300	−4.13532800	2.01511500
C	4.15305700	−4.51931500	0.57323900
H	4.88767200	−3.37484600	−1.10503200
H	3.15626800	−5.49206000	2.21557600
H	5.05604900	−5.12103900	0.66200000
C	−0.48979600	−2.73307100	−1.30963000
C	−1.02175500	−1.94941000	−2.33554200
C	−0.62764600	−4.12506100	−1.36858600
C	−1.71339400	−2.53991800	−3.38906900
H	−0.87785300	−0.86885200	−2.31138000
C	−1.31133400	−4.71488500	−2.42415700
H	−0.19723500	−4.74912100	−0.58399300
C	−1.86128500	−3.92208100	−3.42979600
H	−2.13328900	−1.91997200	−4.17880300
H	−1.41385300	−5.79752100	−2.46546500
H	−2.39852900	−4.38739700	−4.25402300
C	−0.72249300	−2.32059900	1.55441200
C	−1.98137900	−2.91406900	1.43921800
C	−0.28156600	−1.89162600	2.81462300
C	−2.78280700	−3.07991000	2.56677700
H	−2.34398900	−3.24462300	0.46616000
C	−1.08363400	−2.05741800	3.93737300

H	0.69752100	−1.41861300	2.91482000
C	−2.33847800	−2.65196400	3.81336400
H	−3.76135500	−3.54702500	2.46609600
H	−0.72972800	−1.71711200	4.90884200
H	−2.96722700	−2.78405300	4.69204600
C	−3.28053700	0.34106900	2.02300200
C	−4.29419600	−0.06305800	1.16765500
C	−4.09091500	−0.05163000	−0.21524800
C	−2.87628300	0.38462200	−0.74238800
C	−1.84571800	0.77008900	0.09995300
C	−2.06150500	0.72867500	1.47609100
H	−3.40245900	0.32688200	3.10251500
H	−5.24706000	−0.39843300	1.56838600
H	−2.76515200	0.43336200	−1.82419200
H	−0.98332300	1.30559500	−0.33956000
N	−0.99569000	1.09521600	2.39374100
O	0.18554100	1.02602200	2.00056900
O	−1.28369000	1.45016300	3.51576200
C	−5.13877400	−0.47771000	−1.19060900
O	−4.95743800	−0.49905700	−2.38671100
O	−6.27525900	−0.82147000	−0.58792200
C	−7.34845100	−1.23161200	−1.46166500
H	−7.00356000	−2.08991600	−2.05190000
H	−7.55193400	−0.41283500	−2.16304900
C	−8.53277500	−1.56091600	−0.59525600
H	−8.84183400	−0.69021100	−0.00548700
H	−9.37953500	−1.86845500	−1.21882700
H	−8.30086000	−2.38096300	0.09401600

Substrate 12—Position 16 (Nitro Bound Transition State)

1 1

C	0.40622500	−4.66648000	−0.57798800
C	−0.93637900	−4.59029300	−0.72229500
N	0.85607300	−3.37497100	−0.34559300
N	−1.28210900	−3.25733300	−0.56975200
C	−2.62824700	−2.75654900	−0.62190300
C	−3.35785300	−2.70224600	0.57038600
C	−3.11332000	−2.25644800	−1.83195600
C	−4.61169100	−2.09874400	0.52761400
C	−4.36972700	−1.65322600	−1.81887100
C	−5.12993100	−1.56350600	−0.65336300

H	−5.20086000	−2.04076500	1.44460200
H	−4.76548100	−1.24194900	−2.74945300
C	2.23608800	−3.03131900	−0.13466400
C	2.76080300	−3.17306800	1.15579000
C	2.99877100	−2.57173000	−1.21393800
C	4.08405600	−2.78571600	1.35768100
C	4.31658800	−2.19792800	−0.95616200
C	4.87264100	−2.28653400	0.32021400
H	4.50741400	−2.86914800	2.36015900
H	4.92780700	−1.82433300	−1.78051400
C	2.43800100	−2.45514200	−2.60040700
H	2.02154100	−1.45108000	−2.78477700
H	3.22178900	−2.61340700	−3.35028200
H	1.63388200	−3.17675400	−2.79352100
C	1.93609700	−3.72880200	2.27768600
H	0.97916000	−3.19881000	2.38295600
H	1.70295900	−4.79082500	2.11733400
H	2.46994800	−3.64786300	3.23047100
C	6.29252000	−1.87350600	0.56152100
H	6.49081600	−1.71361300	1.62798800
H	6.99447300	−2.64183000	0.20965000
H	6.53627500	−0.94696600	0.02553300
C	−6.49022300	−0.93617500	−0.67216700
H	−7.27651900	−1.69992900	−0.74907200
H	−6.68378700	−0.36617700	0.24550500
H	−6.60704100	−0.25918700	−1.52697900
C	−2.31408400	−2.34738700	−3.09692100
H	−1.38107300	−1.76635200	−3.03776300
H	−2.02721100	−3.38290200	−3.32712000
H	−2.88876100	−1.96610400	−3.94852800
C	−2.78672200	−3.22241200	1.85437700
H	−2.49127300	−4.27810700	1.78077600
H	−1.88570800	−2.66169400	2.15008600
H	−3.51497500	−3.13666600	2.66828600
H	−1.68638900	−5.34656500	−0.91315600
H	1.09232600	−5.50229300	−0.61737800
C	−0.17959900	−2.50014100	−0.33181700
Ir	−0.12913500	−0.43195500	−0.05756200
H	−1.65637800	−0.75322000	0.46600500
H	−0.94639500	−0.28016700	−1.39384400
P	−1.07864200	1.75566200	0.29609900
C	−1.72619300	2.05877300	1.97211300

C	−2.04034300	0.99687200	2.82473200
C	−1.98968000	3.37278600	2.38248700
C	−2.60493300	1.24580400	4.07223200
H	−1.84053400	−0.02803600	2.51470500
C	−2.55614800	3.61516600	3.62730700
H	−1.75570100	4.20948300	1.72417600
C	−2.86216900	2.55190200	4.47369700
H	−2.83847500	0.41379400	4.73347500
H	−2.75844700	4.63809300	3.93834400
H	−3.30116500	2.74389100	5.45088300
C	−2.51861500	1.93295600	−0.82019600
C	−2.34973300	1.74704900	−2.19898700
C	−3.79579100	2.19144900	−0.32039500
C	−3.43329800	1.84366700	−3.06159900
H	−1.35999500	1.52589700	−2.60434100
C	−4.88302400	2.27726000	−1.18781000
H	−3.94887800	2.32961200	0.74920000
C	−4.70479700	2.10818800	−2.55524900
H	−3.28667400	1.70932300	−4.13193400
H	−5.87441300	2.47982500	−0.78598000
H	−5.55568600	2.18126700	−3.23044100
C	0.01621000	3.18548600	−0.01301200
C	−0.04889300	3.95555200	−1.17551100
C	1.02366400	3.44558700	0.92504500
C	0.89061200	4.96055200	−1.40148500
H	−0.84073300	3.78988000	−1.90481100
C	1.95786400	4.44640700	0.69648900
H	1.07696500	2.86017500	1.84505500
C	1.89813400	5.20179100	−0.47343400
H	0.82572200	5.56412900	−2.30542700
H	2.73929200	4.63311000	1.43096100
H	2.63392000	5.98288800	−0.65507900
C	2.50977500	0.35722500	1.06752100
C	1.78115900	0.47320000	−0.12197000
C	2.38748700	1.23296000	−1.13146100
C	3.63988700	1.81402000	−0.95674700
C	4.34440300	1.63605700	0.24753700
C	3.77957500	0.91110600	1.27192100
H	0.83691700	−0.70800000	−1.29690900
H	1.84930700	1.37931700	−2.06957900
H	5.32335900	2.09905500	0.34838400
H	4.27793500	0.76704100	2.22827800

C	4.28196000	2.67083400	−2.01278000
O	5.36737000	3.17141900	−1.79966700
C	3.54615100	2.89575900	−3.30485700
H	3.33560400	1.94664600	−3.81594700
H	2.58219800	3.39178100	−3.12019900
H	4.15898100	3.52606000	−3.95430500
N	1.88858600	−0.33203000	2.15834400
O	2.45292100	−0.49059500	3.21732000
O	0.70914600	−0.75443400	1.97658500

Substrate 12—Position 17 (Nitro Bound C−H Activated product)

1 1

C	1.30272400	−3.84165600	−2.25084700
C	0.13664500	−3.62764000	−2.89789900
N	1.31450200	−2.99243200	−1.15329700
N	−0.53905000	−2.65396400	−2.18227600
C	−1.83606400	−2.15839600	−2.54312300
C	−2.96312000	−2.80211800	−2.01967900
C	−1.92299100	−1.01938700	−3.34901900
C	−4.20770500	−2.23061500	−2.27593800
C	−3.19183600	−0.48800400	−3.57641300
C	−4.34042800	−1.06538500	−3.03509100
H	−5.10211700	−2.71244200	−1.87656500
H	−3.28421700	0.40636200	−4.19533700
C	2.40559400	−2.97650000	−0.21791900
C	2.29036600	−3.72187100	0.96072800
C	3.56327700	−2.26822700	−0.55530400
C	3.36996700	−3.70356600	1.83902800
C	4.61401000	−2.28047300	0.36093900
C	4.53028700	−2.97731000	1.56538200
H	3.29976700	−4.26494100	2.77193800
H	5.52235400	−1.72253100	0.12548500
C	3.68432400	−1.53816300	−1.85881800
H	3.81520300	−2.23349900	−2.70025700
H	2.79141100	−0.93558400	−2.07880300
H	4.55082300	−0.86718900	−1.85088400
C	1.04817300	−4.49487200	1.28302800
H	0.25062600	−3.82800100	1.64251300
H	0.65728000	−5.03551800	0.40981100
H	1.24008900	−5.22752700	2.07450400
C	5.64002900	−2.91314500	2.56766500

H	5.43288100	−2.13226300	3.31352700
H	5.75114800	−3.85878900	3.11168100
H	6.60088700	−2.67435000	2.09695700
C	−5.68890200	−0.44930500	−3.25504100
H	−6.46423600	−1.21329900	−3.38997400
H	−5.98690700	0.16441700	−2.39326400
H	−5.69691500	0.20198000	−4.13652300
C	−0.70388800	−0.39197600	−3.95561400
H	0.15146700	−0.38678200	−3.26658500
H	−0.38186100	−0.94064400	−4.85257900
H	−0.89938700	0.64335200	−4.26197400
C	−2.84204100	−4.07128700	−1.22847500
H	−2.52971000	−4.91096600	−1.86531900
H	−2.09627300	−3.99978200	−0.42367400
H	−3.80203700	−4.34383400	−0.77625300
H	−0.28639700	−4.06544900	−3.79261400
H	2.12848800	−4.51263400	−2.44806800
C	0.17973400	−2.24818500	−1.09414300
Ir	−0.47617000	−0.74009900	0.22304400
H	−1.69767500	−1.94170000	0.96838800
H	−2.09080700	−1.65900800	0.30011900
P	−1.47102800	1.10281600	1.37295900
C	−2.96809400	0.65058600	2.32588500
C	−4.00626800	0.03709300	1.61317400
C	−3.13988000	0.92894900	3.68260600
C	−5.18769400	−0.31069800	2.25303000
H	−3.89523500	−0.15160700	0.54193200
C	−4.32537400	0.57349900	4.32212400
H	−2.35260300	1.43003600	4.24450400
C	−5.34513900	−0.04966700	3.61307400
H	−5.98995100	−0.78499500	1.69067700
H	−4.45048500	0.79054000	5.38119600
H	−6.26891100	−0.32614100	4.11742700
C	−2.10216300	2.45871300	0.31850000
C	−1.99058900	2.45178600	−1.07242800
C	−2.75216900	3.53126400	0.94486300
C	−2.50060300	3.50599400	−1.82591600
H	−1.51108300	1.61610300	−1.57850500
C	−3.25997900	4.58116600	0.19252600
H	−2.85962100	3.54422600	2.03028600
C	−3.13168300	4.57102700	−1.19529000
H	−2.39667900	3.49139000	−2.90950500

H	−3.76157000	5.40912900	0.68966000
H	−3.52928700	5.39554500	−1.78388700
C	−0.30633300	1.89911000	2.52638200
C	0.27302900	3.13627800	2.22712200
C	0.14039100	1.18487300	3.64617200
C	1.27052700	3.65638100	3.04570700
H	−0.05080000	3.69396400	1.34880900
C	1.13109700	1.71321200	4.46537400
H	−0.27331300	0.20030300	3.86860100
C	1.69769800	2.94939700	4.16516400
H	1.71347900	4.62156000	2.80742100
H	1.46860800	1.15022900	5.33327800
H	2.47639400	3.35977300	4.80511500
C	3.33340500	1.12029000	0.75525500
C	3.48859000	2.01674300	−0.28339200
C	2.48071600	2.11880300	−1.25393400
C	1.33319100	1.33406200	−1.18228800
C	1.12824100	0.42295500	−0.14206200
C	2.16805500	0.35205300	0.80129400
H	4.08511100	0.99967900	1.53137700
H	4.37515500	2.64009700	−0.35546500
H	0.58798500	1.46925700	−1.96326700
H	−1.29001900	−0.23409600	−1.02073700
N	1.99336000	−0.55937800	1.89961600
O	0.92423500	−1.22595800	1.92879100
O	2.83332000	−0.67842400	2.76344700
C	2.57744300	3.06062400	−2.40922500
O	1.73466500	3.14447000	−3.27478600
O	3.69332000	3.79005700	−2.37242300
C	3.86493100	4.72700200	−3.45372400
H	2.99675400	5.39788400	−3.47049800
H	3.86469800	4.16845200	−4.39845000
C	5.15771900	5.45782000	−3.21418900
H	6.00539100	4.76310200	−3.19849800
H	5.32960800	6.18904900	−4.01190600
H	5.13364300	5.99441200	−2.25863000

Substrate 12—Position 15 (Ester Bound Reactant)

1 1

C	−0.40952000	−4.63890100	−0.28308100
C	0.93416900	−4.66810400	−0.14591900

N	−0.76833200	−3.30345300	−0.41873400
N	1.36735200	−3.35246400	−0.20206500
C	2.73394800	−2.93904000	−0.05201900
C	3.52145600	−2.79842900	−1.19820800
C	3.19449600	−2.60147800	1.22659200
C	4.80332700	−2.27200200	−1.03925900
C	4.48398200	−2.09139500	1.33208100
C	5.29650600	−1.90252500	0.21137300
H	5.43246100	−2.14232300	−1.92130700
H	4.86730300	−1.81848500	2.31737700
C	−2.13382900	−2.88365600	−0.54982700
C	−2.65468400	−2.67255300	−1.83247100
C	−2.91189800	−2.75649000	0.60650200
C	−3.99908600	−2.32073800	−1.93343100
C	−4.25186200	−2.40421900	0.45122500
C	−4.81394100	−2.18862700	−0.80571800
H	−4.42675200	−2.14561100	−2.92247700
H	−4.87137800	−2.28314800	1.34249700
C	−2.34262500	−3.00116500	1.97294900
H	−2.37685100	−4.06767100	2.23843400
H	−1.29063400	−2.69223000	2.04654600
H	−2.91816600	−2.46087400	2.73624600
C	−1.79169300	−2.81401000	−3.04991900
H	−0.96341700	−2.09022700	−3.04296400
H	−1.33819200	−3.81335000	−3.11300700
H	−2.37371800	−2.65326200	−3.96380000
C	−6.26174300	−1.82809200	−0.94637800
H	−6.43698100	−1.18928800	−1.82092900
H	−6.88106400	−2.72646800	−1.07460600
H	−6.63756600	−1.30688200	−0.05549600
C	6.66485900	−1.31245300	0.36605000
H	7.16048100	−1.17856300	−0.60196700
H	6.61478700	−0.33112700	0.85757100
H	7.30801300	−1.95023500	0.98699900
C	2.30878500	−2.71573100	2.43088600
H	1.47674800	−1.99502100	2.37870600
H	1.86020200	−3.71415000	2.52815000
H	2.87368300	−2.51133600	3.34847000
C	2.98091500	−3.13083200	−2.55514800
H	2.56168300	−4.14498700	−2.59909300
H	2.17011000	−2.43994800	−2.83442600
H	3.76333700	−3.05636500	−3.31827800

H	1.63015100	−5.48669500	−0.01671800
H	−1.15197100	−5.42600000	−0.30345900
C	0.32292800	−2.49073900	−0.37133600
Ir	0.54312800	−0.43410500	−0.44280400
H	0.81387700	−0.54236700	−1.98376800
H	2.05956600	−0.72969600	−0.54809600
P	1.23120800	1.82232900	−0.47607300
C	1.93652800	2.44118100	−2.04402300
C	2.78502700	1.58608000	−2.75731600
C	1.72582900	3.74193800	−2.51000500
C	3.40562400	2.02565200	−3.91987500
H	2.96419500	0.57146600	−2.39608100
C	2.34140000	4.17439400	−3.68090700
H	1.07795800	4.42335200	−1.95865100
C	3.17928800	3.31787700	−4.38688900
H	4.06379800	1.35486400	−4.46891400
H	2.16583600	5.18651700	−4.04043600
H	3.65844000	3.65784300	−5.30295900
C	2.57112600	2.12549800	0.73772100
C	2.82442600	1.19458000	1.74923000
C	3.32259600	3.30483400	0.69049800
C	3.80875600	1.44216800	2.70263400
H	2.24797100	0.26995200	1.78664500
C	4.30674400	3.54802500	1.64079500
H	3.14001700	4.03700900	−0.09692600
C	4.55026900	2.61759200	2.64906500
H	3.99737000	0.70862800	3.48624100
H	4.88842100	4.46668100	1.59476800
H	5.32201000	2.81010800	3.39216700
C	−0.06682000	3.01875900	0.00369100
C	−0.11232500	3.57254400	1.28697100
C	−1.12150100	3.26805500	−0.88319700
C	−1.19271700	4.36322500	1.67220400
H	0.70439000	3.39513200	1.98805100
C	−2.19118100	4.06786500	−0.50056200
H	−1.10451400	2.83075700	−1.88401400
C	−2.23026600	4.61446500	0.78082500
H	−1.21524700	4.79526400	2.67118200
H	−3.00024300	4.26071000	−1.20379600
H	−3.07056200	5.23779900	1.08100100
C	−4.69431700	1.45381700	1.21303500
C	−3.58130200	1.12520000	1.97238300

C	−2.43384000	0.60698800	1.35873100
C	−2.41758800	0.43141000	−0.02692600
C	−3.51625000	0.76470800	−0.80119600
C	−4.63975700	1.26358400	−0.16064300
H	−5.59977300	1.84908000	1.66442500
H	−3.59657300	1.26883900	3.04902500
H	−1.56063600	0.00096500	−0.56152300
H	−3.51536600	0.63005700	−1.87906400
N	−5.82976700	1.58119700	−0.97156100
O	−5.74904000	1.39379300	−2.17399500
O	−6.81117100	1.99456400	−0.38260700
C	−1.25916300	0.22386200	2.17422400
O	−0.20832200	−0.23369400	1.72500400
O	−1.43240400	0.39290600	3.46949200
C	−0.32831700	0.02664600	4.33078500
H	0.56318300	0.57740700	3.99933900
H	−0.13495400	−1.04596700	4.19004900
C	−0.73082000	0.36152000	5.73989300
H	−1.62709700	−0.19461500	6.03652300
H	0.07842400	0.09757400	6.42950400
H	−0.93464100	1.43278400	5.84669400

Substrate 12—Position 16 (Ester Bound Transition State)

1 1

C	1.04591000	−4.04666400	−1.79856100
C	−0.25835000	−4.33674400	−1.60820500
N	1.22559200	−2.73310900	−1.38673300
N	−0.84969600	−3.19631900	−1.08755400
C	−2.25054600	−3.13266200	−0.77073800
C	−2.65737400	−3.42780000	0.53444500
C	−3.15014300	−2.76589500	−1.77740400
C	−4.01446200	−3.31223000	0.82952600
C	−4.49631500	−2.66681700	−1.43170400
C	−4.94649100	−2.93386500	−0.13718200
H	−4.35282600	−3.52806600	1.84423200
H	−5.21653400	−2.37803100	−2.19913900
C	2.50602700	−2.08810100	−1.44098800
C	3.44471700	−2.38090200	−0.44570400
C	2.78938500	−1.24242700	−2.52018000
C	4.67316200	−1.72286900	−0.50618100
C	4.03602900	−0.61976700	−2.53853700

C	4.97929600	−0.82721800	−1.52976700
H	5.41278400	−1.91766500	0.27272700
H	4.28497100	0.03801800	−3.37532700
C	1.79384600	−1.01510400	−3.61721800
H	1.35752700	−1.95770100	−3.97550300
H	0.96107600	−0.38208000	−3.27779800
H	2.26604700	−0.51814400	−4.47296800
C	3.16832100	−3.38156600	0.63696600
H	2.14340300	−3.31237700	1.02668200
H	3.30194900	−4.41010600	0.27191300
H	3.85866100	−3.24766600	1.47845000
C	6.27672500	−0.07866000	−1.53741500
H	7.04921000	−0.59239900	−0.95371300
H	6.65884000	0.06232000	−2.55628100
H	6.14750000	0.92295300	−1.09967200
C	−6.40572100	−2.85053000	0.19613800
H	−6.56669900	−2.69537200	1.27030300
H	−6.90132100	−2.03707400	−0.34951800
H	−6.92652800	−3.77898200	−0.07615000
C	−2.68496500	−2.47835000	−3.17207600
H	−3.52615200	−2.19190300	−3.81249100
H	−1.95245100	−1.65801200	−3.18865800
H	−2.20033900	−3.35142400	−3.63110700
C	−1.66614900	−3.82135300	1.58632000
H	−1.02402100	−4.65134200	1.26103300
H	−0.99718500	−2.98355100	1.83387600
H	−2.17104400	−4.12736700	2.50869700
H	−0.83351000	−5.23542100	−1.78905100
H	1.87037900	−4.63203600	−2.18393000
C	0.06014000	−2.18880500	−0.94239300
Ir	−0.42974300	−0.26016600	−0.26141100
H	−1.70857400	−1.02497300	0.23260400
H	−1.42298600	−0.20972600	−1.56525900
P	−1.43317600	1.82811900	0.27795300
C	−2.97051300	1.66871000	1.24435100
C	−3.89297200	0.68815800	0.86002900
C	−3.27997000	2.52700900	2.30351300
C	−5.10230700	0.56527200	1.53274700
H	−3.66761900	0.01933400	0.02770100
C	−4.49121600	2.39689800	2.97626600
H	−2.57218200	3.29797500	2.60839100
C	−5.40121800	1.41670900	2.59305000

H	−5.80968100	−0.20494500	1.22663000
H	−4.72351200	3.06491600	3.80329900
H	−6.34690200	1.31655600	3.12231300
C	−1.87854600	2.89688900	−1.13495200
C	−1.35084100	2.66992800	−2.40929700
C	−2.72424600	3.99257900	−0.92506200
C	−1.66636300	3.52864600	−3.45812800
H	−0.69080000	1.82141300	−2.58170200
C	−3.03850800	4.84498900	−1.97603000
H	−3.14805300	4.17618800	0.06267600
C	−2.50982800	4.61366100	−3.24328900
H	−1.25677900	3.34367500	−4.44959600
H	−3.70256300	5.69007500	−1.80602200
H	−2.76095200	5.27959000	−4.06670400
C	−0.28332100	2.83625800	1.27677700
C	0.43260600	3.88854700	0.69947300
C	0.03303100	2.43021800	2.58057200
C	1.44985300	4.52070200	1.41155300
H	0.18970500	4.22035700	−0.31069100
C	1.04168900	3.06756200	3.29075300
H	−0.51308500	1.60737700	3.04583300
C	1.75684600	4.11081800	2.70377000
H	1.99729200	5.34275200	0.95339100
H	1.27368700	2.74865100	4.30535000
H	2.54972800	4.60776600	3.25950900
C	3.61471400	0.11350200	2.69801600
C	3.40573700	0.84826800	1.54164700
C	2.24952700	0.65319800	0.78294000
C	1.23326000	−0.25453900	1.17152600
C	1.48145100	−1.01165100	2.31753200
C	2.65375700	−0.82738200	3.03806000
H	4.50138800	0.23217700	3.31327100
H	4.15039000	1.56455700	1.20026800
H	−0.38484300	−0.36795300	1.34097000
H	0.77156500	−1.75416600	2.67849100
N	2.89396600	−1.69021300	4.21187200
O	2.15344700	−2.64775100	4.35832100
O	3.82244600	−1.39273900	4.94075600
C	2.09621300	1.29523300	−0.52255300
O	1.13730400	1.02818900	−1.26295600
O	3.04148900	2.12975300	−0.89070500
C	2.88516300	2.75791800	−2.18394900

H	2.01555200	3.42888800	−2.12828500
H	2.66006500	1.97539900	−2.91998100
C	4.16765800	3.48054300	−2.48522700
H	5.00394300	2.77289200	−2.54420900
H	4.08942400	3.99739700	−3.44801100
H	4.39520900	4.22430900	−1.71331100

Substrate 12—Position 17 (Ester Bound C−H Activated Product)

1 1

C	1.17639400	−4.30903200	−0.93467400
C	−0.14610400	−4.55419900	−0.82445800
N	1.33974300	−2.93456300	−0.82722000
N	−0.76435700	−3.32594200	−0.65197200
C	−2.18855600	−3.18579700	−0.53029600
C	−2.77836400	−3.15928400	0.73525700
C	−2.93846600	−3.07448100	−1.71175000
C	−4.16177400	−2.96959500	0.79726600
C	−4.30909100	−2.87197000	−1.59372000
C	−4.93851700	−2.80554500	−0.34612500
H	−4.64111500	−2.94934300	1.77868000
H	−4.90917100	−2.77746700	−2.50031300
C	2.63096700	−2.31618100	−0.93458300
C	3.51930200	−2.42560400	0.14240300
C	2.97975600	−1.69969600	−2.14122900
C	4.76509300	−1.81545500	0.01097300
C	4.23843900	−1.10530900	−2.21855700
C	5.13433200	−1.13434500	−1.14876800
H	5.46689000	−1.87090200	0.84528700
H	4.53609700	−0.61901000	−3.15058400
C	2.04333300	−1.67656800	−3.31170800
H	1.55474300	−2.64879300	−3.46578700
H	1.25213500	−0.92549800	−3.16906500
H	2.58146600	−1.42220500	−4.23237800
C	3.17031200	−3.19002700	1.38470800
H	2.14339800	−2.99732900	1.72440100
H	3.25532900	−4.27458800	1.22571700
H	3.85081500	−2.93187300	2.20484400
C	6.45267300	−0.42839700	−1.23365800
H	7.23827800	−0.96345900	−0.68684900
H	6.78343200	−0.30773500	−2.27230300
H	6.38166000	0.57727500	−0.79338900

C	−6.41292200	−2.55514700	−0.25237700
H	−6.97062700	−3.15184000	−0.98470200
H	−6.80162600	−2.79352700	0.74459000
H	−6.64597800	−1.50022500	−0.45595700
C	−2.28545100	−3.17230300	−3.05838300
H	−1.46523900	−2.44862100	−3.17760200
H	−1.84791800	−4.16641400	−3.22580100
H	−3.01178800	−2.99250200	−3.85825600
C	−1.99223500	−3.29020700	2.00504500
H	−0.96605000	−3.63973100	1.83898200
H	−1.93321200	−2.32272300	2.52715000
H	−2.47990100	−3.99027500	2.69443500
H	−0.71707300	−5.47318200	−0.84465400
H	2.02335800	−4.96572900	−1.08335600
C	0.14517000	−2.30549200	−0.65607000
Ir	−0.39453800	−0.29956000	−0.37314000
H	−1.94192500	−0.64419800	−1.37302800
H	−1.41360200	−0.38582600	−1.94959600
P	−1.34347100	1.84133200	0.11744400
C	−2.74421000	1.77834100	1.28739200
C	−3.58332500	0.65857500	1.28561300
C	−3.05823000	2.86362700	2.11338100
C	−4.70494600	0.61811600	2.10558900
H	−3.36434300	−0.19303900	0.64004700
C	−4.18089400	2.81914200	2.93358800
H	−2.42470900	3.75068200	2.11541300
C	−5.00339200	1.69633600	2.93304600
H	−5.34315200	−0.26508100	2.09799100
H	−4.41402400	3.66673800	3.57499000
H	−5.87921200	1.66407100	3.57821500
C	−2.02365200	2.67882200	−1.36349900
C	−1.35426200	2.55811400	−2.58934000
C	−3.18807300	3.44950800	−1.29460100
C	−1.84225300	3.20379300	−3.72038300
H	−0.44021300	1.96601100	−2.65448100
C	−3.67477000	4.08781100	−2.43090300
H	−3.72556000	3.54776100	−0.35217200
C	−3.00424400	3.96655000	−3.64319900
H	−1.31623600	3.10531200	−4.66842800
H	−4.58454300	4.68155000	−2.36725200
H	−3.38880700	4.46480600	−4.53103500
C	−0.14864800	3.02044000	0.83258200

C	0.51057200	3.95388700	0.02829000
C	0.22967400	2.88343000	2.17401000
C	1.54211800	4.72804500	0.55385300
H	0.20841900	4.08827500	−1.01085800
C	1.24712900	3.66945000	2.69858200
H	−0.27414700	2.15736300	2.81298600
C	1.91299900	4.58576100	1.88632800
H	2.04659700	5.45543400	−0.08027400
H	1.52749200	3.56088000	3.74488200
H	2.71435300	5.19628100	2.29797300
C	3.24070000	0.47793400	2.75213200
C	3.24586900	0.98635900	1.46358900
C	2.17789000	0.71023000	0.60556500
C	1.05458600	−0.04890900	1.00768100
C	1.06878800	−0.56100900	2.30695500
C	2.15263700	−0.30043500	3.13314200
H	4.05246500	0.65300600	3.45121300
H	4.08127900	1.58710400	1.10827100
H	−1.35747000	−0.93001300	0.68709500
H	0.25597700	−1.17783400	2.68332600
N	2.17454400	−0.92743200	4.47016100
O	1.33151700	−1.77830300	4.70141100
O	3.04162900	−0.56467200	5.24479300
C	2.15866100	1.16428100	−0.78279300
O	1.23304700	0.85172500	−1.55151300
O	3.16666500	1.91399000	−1.17380700
C	3.13372700	2.40547700	−2.52949000
H	2.22697500	3.01558800	−2.64752000
H	3.05167200	1.54691000	−3.20988900
C	4.39362400	3.19537100	−2.74892200
H	5.28037700	2.55962800	−2.63903800
H	4.40070100	3.61751100	−3.75983700
H	4.46860700	4.02005300	−2.03074100

Substrate 14—Position 15 (Ketone Bound Reactant)

1 1

C	−0.33391300	−4.60848000	0.08449600
C	0.97572000	−4.62509000	−0.24579000
N	−0.68618800	−3.27482800	0.24389000
N	1.39378800	−3.30528800	−0.28622300
C	2.74683500	−2.91795900	−0.57230400

C	3.10953100	−2.66285600	−1.89618900
C	3.64095200	−2.78097100	0.49659100
C	4.41504000	−2.23074800	−2.13510600
C	4.93140700	−2.34750900	0.20537400
C	5.33474400	−2.06093200	−1.10135800
H	4.72082000	−2.02691000	−3.16292300
H	5.64710100	−2.23250200	1.02136600
C	−2.01316000	−2.87592100	0.61242500
C	−2.98440800	−2.77335800	−0.38948800
C	−2.30089100	−2.66206400	1.96492400
C	−4.27695700	−2.41688700	−0.00646400
C	−3.60757700	−2.30593500	2.29480200
C	−4.60625100	−2.17589600	1.32676300
H	−5.04423300	−2.31140800	−0.77634000
H	−3.85747500	−2.14060300	3.34554800
C	−1.24312000	−2.78970700	3.01856600
H	−0.71357000	−3.75024000	2.95251100
H	−0.48639000	−1.99682700	2.91998400
H	−1.67970800	−2.71386700	4.02105500
C	−2.65098000	−3.04672300	−1.82510300
H	−1.72729800	−2.53841000	−2.13859200
H	−2.49077900	−4.11944000	−2.00322300
H	−3.46353400	−2.72253000	−2.48555900
C	−6.00388300	−1.80103600	1.71738600
H	−6.51452600	−1.25370000	0.91500400
H	−6.60775700	−2.69245700	1.93589800
H	−6.01810500	−1.17751800	2.62116200
C	6.72751300	−1.57807300	−1.37299400
H	6.93658000	−1.52753000	−2.44793000
H	6.88778600	−0.57544900	−0.95232800
H	7.47623500	−2.23786100	−0.91631600
C	3.21837100	−3.06604200	1.90691000
H	2.41018500	−2.39152500	2.22995100
H	2.84215900	−4.09164800	2.02599100
H	4.05740000	−2.93724200	2.59920200
C	2.12616800	−2.81490100	−3.01667000
H	1.63914500	−3.79953700	−3.00775400
H	1.32548800	−2.06317200	−2.94662600
H	2.61604000	−2.69062700	−3.98849300
H	1.65767300	−5.43887700	−0.45556000
H	−1.05560600	−5.40201500	0.22866900
C	0.37399900	−2.44759100	0.01771800

Ir	0.58989600	−0.38635600	0.09987000
H	1.40553000	−0.55933700	−1.22068100
H	2.05940400	−0.68753700	0.49449500
P	1.25323700	1.87369500	0.15553200
C	2.42522600	2.38094700	−1.15109700
C	3.41003700	1.46776200	−1.54676400
C	2.40696900	3.65657900	−1.72406400
C	4.35119700	1.82271200	−2.50537700
H	3.44266000	0.46891800	−1.10841100
C	3.34825400	4.00533600	−2.68776400
H	1.64958200	4.38178400	−1.42570000
C	4.31863100	3.08953300	−3.08160100
H	5.10588000	1.09904400	−2.81053800
H	3.32021300	4.99774900	−3.13362300
H	5.04984600	3.36382000	−3.83950400
C	2.07996000	2.34426400	1.71707500
C	2.03140900	1.48420300	2.81856800
C	2.73037800	3.57737500	1.83688000
C	2.62055700	1.85591100	4.02424500
H	1.53267800	0.51768100	2.72914400
C	3.31617400	3.94558500	3.04124000
H	2.78242000	4.25279500	0.98232600
C	3.26047500	3.08535800	4.13599400
H	2.58326600	1.18024500	4.87683000
H	3.82050600	4.90620500	3.12676100
H	3.72255100	3.37517400	5.07784600
C	−0.16683900	3.02223600	0.03789300
C	−0.71181200	3.61201100	1.18236500
C	−0.82680500	3.17418500	−1.18772800
C	−1.89232600	4.34804700	1.09910900
H	−0.20766100	3.50189600	2.14391700
C	−1.99861000	3.91641800	−1.26935100
H	−0.42065600	2.70818900	−2.08784400
C	−2.53861600	4.49814400	−0.12377200
H	−2.30091100	4.81505400	1.99448300
H	−2.49571100	4.03805200	−2.23067400
H	−3.45627700	5.08006100	−0.18871600
C	−4.47981400	0.99126500	−1.75851000
C	−4.99921500	1.41157000	−0.54238400
C	−4.20054600	1.30729900	0.58665200
C	−2.89988000	0.79331200	0.50218100
C	−2.40778300	0.40445700	−0.74845000

C	−3.19026800	0.49640900	−1.88765600
H	−6.01119100	1.80328500	−0.49563700
H	−4.60460300	1.62570000	1.54517600
H	−1.38663300	0.01974700	−0.87000200
H	−2.82403100	0.18956900	−2.86377500
C	−2.08057900	0.61553100	1.72349400
O	−0.95361100	0.11497200	1.68347200
C	−2.63100900	1.02374900	3.05456800
H	−2.89852400	2.08862400	3.05934700
H	−3.53844000	0.45101000	3.29070100
H	−1.87766000	0.83766100	3.82550100
N	−5.32947700	1.07243600	−2.96281200
O	−4.86422700	0.63066900	−3.99823400
O	−6.43254600	1.56999600	−2.83204800

Substrate 14—Position 16 (Ketone Bound Transition State)

1 1

C	1.80535000	−3.91934300	−1.20978800
C	0.58290000	−4.37979700	−0.87297200
N	1.76551000	−2.53935400	−1.06152900
N	−0.18184600	−3.27508500	−0.53167000
C	−1.57704400	−3.39924100	−0.20476500
C	−1.96655600	−3.51781800	1.13122800
C	−2.49585800	−3.42076100	−1.26387000
C	−3.33208000	−3.64728700	1.39544700
C	−3.84358700	−3.54900500	−0.94621800
C	−4.28142300	−3.66722200	0.37627400
H	−3.65754600	−3.74443400	2.43285400
H	−4.57650700	−3.56818700	−1.75467700
C	2.88866300	−1.70637800	−1.38096100
C	3.98537700	−1.68865100	−0.50883500
C	2.87301200	−0.99715600	−2.58562100
C	5.05601300	−0.86150200	−0.83917200
C	3.96786400	−0.17894600	−2.86472600
C	5.06053400	−0.09001900	−2.00244700
H	5.91562000	−0.82129900	−0.16688200
H	3.97234500	0.39228300	−3.79546200
C	1.73449700	−1.11563200	−3.55302500
H	1.44856200	−2.16456000	−3.71527700
H	0.83998200	−0.58814300	−3.19251800
H	2.00599500	−0.68655800	−4.52403400

C	4.03164200	−2.54335100	0.72278000
H	3.08352500	−2.53543000	1.27837500
H	4.24564000	−3.59297500	0.47589300
H	4.82756600	−2.20991600	1.39948900
C	6.22671700	0.79701600	−2.31958000
H	7.11336700	0.20879400	−2.59189900
H	6.00975800	1.46888400	−3.15884900
H	6.51014200	1.41088000	−1.45343700
C	−5.74042800	−3.83530500	0.67656400
H	−6.34363600	−3.06313500	0.18073600
H	−6.10977200	−4.80498100	0.31647400
H	−5.94139900	−3.78666900	1.75347100
C	−2.04331000	−3.28883400	−2.68587100
H	−1.58903300	−2.30246100	−2.86465000
H	−1.28785200	−4.04150700	−2.95102900
H	−2.88530100	−3.40141800	−3.37720800
C	−0.97897100	−3.45768100	2.25647900
H	−0.01252600	−3.90941100	1.99758800
H	−0.78262500	−2.41303900	2.54433300
H	−1.36470600	−3.96874200	3.14552600
H	0.17030400	−5.37956300	−0.83863300
H	2.70178100	−4.42424800	−1.54469900
C	0.53816700	−2.12167100	−0.64534400
Ir	−0.25695600	−0.21115500	−0.27217900
H	−1.33183100	−1.10705400	0.43765700
H	−1.29487200	−0.57401300	−1.49236200
P	−1.66048700	1.68613200	0.03830900
C	−2.99763700	1.37310600	1.24078100
C	−3.66689900	0.14394200	1.19096600
C	−3.40055000	2.33510800	2.17246500
C	−4.70843000	−0.12106600	2.07089300
H	−3.37577100	−0.61246600	0.45989600
C	−4.44480500	2.06385300	3.05148900
H	−2.89515200	3.30002200	2.21810400
C	−5.09647400	0.83567100	3.00503700
H	−5.21086300	−1.08659100	2.02756500
H	−4.74789000	2.81639800	3.77675200
H	−5.90807900	0.62422200	3.69844900
C	−2.49085500	2.33524200	−1.44847500
C	−1.99802100	2.04829300	−2.72447200
C	−3.59586700	3.18261900	−1.30821400
C	−2.60516200	2.60502600	−3.84583900

H	−1.13659500	1.39226300	−2.83965500
C	−4.20133100	3.73150100	−2.43183300
H	−3.99031300	3.40904300	−0.31740300
C	−3.70585400	3.44343500	−3.70082500
H	−2.21878700	2.37781300	−4.83762300
H	−5.06410300	4.38443500	−2.31657900
H	−4.18208500	3.87317600	−4.58006600
C	−0.70270100	3.09809600	0.69477600
C	−0.38119100	4.18724100	−0.11912100
C	−0.14940100	3.01888600	1.98059400
C	0.47304300	5.18451200	0.34850800
H	−0.80058600	4.26034800	−1.12295700
C	0.69985700	4.01457000	2.44448900
H	−0.38750600	2.17335200	2.62829600
C	1.01738500	5.09748800	1.62525000
H	0.70715300	6.03579200	−0.28872300
H	1.11654500	3.94576900	3.44779000
H	1.68264700	5.87802300	1.98961300
C	3.11096600	0.16029100	2.71270800
C	3.89022900	1.16751300	2.16107700
C	3.46593500	1.71636000	0.96248800
C	2.28094700	1.28345500	0.35373100
C	1.46669100	0.28202100	0.94997000
C	1.92557600	−0.28019000	2.14297000
H	4.80543500	1.47949400	2.65503100
H	4.07927100	2.47545400	0.47919800
H	−0.11518800	0.01282200	1.31475800
H	1.37177800	−1.06884100	2.64968400
C	1.89524000	1.76534200	−0.96975500
O	0.94240700	1.21394800	−1.55159700
C	2.61017100	2.88686600	−1.64604700
H	2.58743200	3.78609400	−1.01482700
H	3.66298300	2.62885400	−1.82520400
H	2.12806400	3.10169100	−2.60399300
N	3.57913800	−0.49653200	3.95127500
O	2.94925700	−1.46454900	4.34055200
O	4.56882500	−0.03190600	4.48637200

Substrate 14—Position 17 (Ketone Bound C−H Activated Product)

1 1

C	1.61237600	−4.27065600	−0.44674200

C	0.33713200	−4.60393800	−0.15722300
N	1.65065100	−2.88755300	−0.54907700
N	−0.37564400	−3.41705700	−0.08341300
C	−1.77547200	−3.37411100	0.23317500
C	−2.16347600	−3.20737500	1.56532900
C	−2.70053000	−3.50875400	−0.81050400
C	−3.53049000	−3.11279400	1.82988300
C	−4.05149000	−3.39166300	−0.49560200
C	−4.48512200	−3.17908700	0.81623600
H	−3.85605100	−2.98915400	2.86439800
H	−4.79028000	−3.48375400	−1.29362300
C	2.88169000	−2.18468900	−0.76852600
C	3.72357900	−1.97716500	0.33070000
C	3.21703100	−1.79339900	−2.06672600
C	4.91403100	−1.29037400	0.10565300
C	4.42261000	−1.11279900	−2.23680700
C	5.27490800	−0.84218800	−1.16587000
H	5.57603800	−1.09208800	0.95100100
H	4.70704700	−0.79188700	−3.24131600
C	2.31803300	−2.08851700	−3.22877300
H	2.10834600	−3.16377400	−3.31648300
H	1.35210800	−1.57243000	−3.12910600
H	2.77622000	−1.76010700	−4.16887500
C	3.36938600	−2.48705200	1.69601100
H	2.31189800	−2.31915100	1.94459700
H	3.54868600	−3.56900400	1.77636100
H	3.97670800	−2.00121200	2.46870900
C	6.55729000	−0.09398400	−1.37029600
H	6.69941500	0.67378000	−0.59766300
H	7.42512400	−0.76491100	−1.31332100
H	6.58944100	0.39668600	−2.35086600
C	−5.94483600	−3.02770400	1.11942900
H	−6.14361300	−3.09601000	2.19535500
H	−6.32074100	−2.05477100	0.77255400
H	−6.54280100	−3.79760300	0.61599500
C	−2.25270900	−3.77446700	−2.21634800
H	−1.48445300	−3.06265900	−2.55356500
H	−1.80862800	−4.77501200	−2.31375400
H	−3.09541000	−3.71800300	−2.91376200
C	−1.16030900	−3.11617700	2.67459000
H	−0.34202800	−3.83855000	2.55455900
H	−0.70126000	−2.11721100	2.71807500

H	−1.63383700	−3.29850600	3.64547600
H	−0.14202400	−5.55960000	0.01141700
H	2.50360300	−4.86739900	−0.59046400
C	0.42629600	−2.33778100	−0.33188400
Ir	−0.27866500	−0.36046500	−0.36050000
H	−1.91548200	−1.06203300	−0.93205600
H	−1.53308700	−0.89962800	−1.64392900
P	−1.42702100	1.73458100	−0.19146100
C	−2.61694500	1.82689600	1.19004000
C	−3.33634300	0.67993000	1.54636200
C	−2.91903900	3.04294500	1.81307400
C	−4.33073700	0.74956400	2.51548800
H	−3.12596500	−0.27812800	1.06664900
C	−3.90692200	3.10505100	2.79040100
H	−2.38302500	3.94911900	1.53197000
C	−4.61302400	1.95940500	3.14305600
H	−4.88649000	−0.14772800	2.78403500
H	−4.12660100	4.05440800	3.27480700
H	−5.38637600	2.01115100	3.90690500
C	−2.44263600	2.09740200	−1.67218800
C	−1.95893600	1.74108600	−2.93816600
C	−3.68183500	2.73646100	−1.56790500
C	−2.70227700	2.02767800	−4.07806400
H	−0.99017000	1.24882600	−3.03283900
C	−4.42359500	3.01463800	−2.71142400
H	−4.07615100	3.01276600	−0.59081000
C	−3.93650700	2.66142400	−3.96557900
H	−2.31746600	1.74953800	−5.05752300
H	−5.38909400	3.50827700	−2.61886800
H	−4.52009700	2.88074400	−4.85759700
C	−0.32082600	3.17465100	−0.00251200
C	0.05926600	3.93086400	−1.11368800
C	0.26714700	3.44119000	1.24074200
C	1.01652200	4.93533100	−0.98433100
H	−0.40039100	3.74601400	−2.08499000
C	1.21924900	4.44451400	1.36625900
H	−0.02401300	2.86393900	2.11884600
C	1.60108800	5.18908500	0.25171600
H	1.29359100	5.53018400	−1.85354800
H	1.66818500	4.64257300	2.33821600
H	2.34621400	5.97598400	0.35258300
C	2.71630400	0.99394500	2.46736600

C	3.59270300	1.73871600	1.68524100
C	3.34361700	1.79463500	0.32539000
C	2.23550700	1.13160800	−0.21999800
C	1.32082700	0.40841600	0.59307500
C	1.59894500	0.34637100	1.96142600
H	4.43995400	2.23772500	2.14558200
H	4.01505100	2.36199200	−0.31837800
H	−0.96186400	−0.78495100	0.99319300
H	0.95655000	−0.19847600	2.64915500
C	1.95217300	1.13902000	−1.64855900
O	0.95459600	0.51387400	−2.06652900
C	2.79014200	1.89219100	−2.62753100
H	2.65001500	2.97200600	−2.47367300
H	3.85643100	1.67458100	−2.49441600
H	2.48903600	1.63327400	−3.64704200
N	3.00190800	0.86809300	3.91157700
O	2.35062000	0.05114200	4.54039700
O	3.87591500	1.58033500	4.37268800

Substrate 14—Position 15 (Nitro Bound Reactant)

1 1

C	−1.41596500	−4.58130400	−0.59248200
C	−2.66926200	−4.07680900	−0.56490400
N	−0.55527300	−3.50536000	−0.42709700
N	−2.54744900	−2.70740400	−0.38442500
C	−3.67642500	−1.82661200	−0.26939500
C	−4.18140200	−1.55528900	1.00842500
C	−4.22203800	−1.27509300	−1.43108800
C	−5.26846900	−0.69147300	1.09918500
C	−5.31231800	−0.41666600	−1.28502100
C	−5.84680100	−0.11424500	−0.03355000
H	−5.68118200	−0.46483400	2.08382300
H	−5.75970400	0.02274700	−2.17875700
C	0.87168900	−3.61400500	−0.38128000
C	1.48199600	−3.89026100	0.84901100
C	1.59989500	−3.41478000	−1.55938900
C	2.87495800	−3.91863400	0.88267100
C	2.99068000	−3.46918800	−1.47349100
C	3.64358100	−3.69774800	−0.26193400
H	3.37456200	−4.11759300	1.83289600
H	3.58136300	−3.30868000	−2.37730400

C	0.91345200	−3.09951200	−2.85442200
H	0.13242800	−3.83289500	−3.09695900
H	0.41773900	−2.11623000	−2.81569700
H	1.62920000	−3.08319600	−3.68356100
C	0.66831000	−4.14212900	2.08284500
H	−0.08418700	−3.35740400	2.24635700
H	0.12604100	−5.09590500	2.01787500
H	1.30826800	−4.18507900	2.97112000
C	5.13806300	−3.64547700	−0.17635300
H	5.46680100	−2.64378000	0.14036000
H	5.53113600	−4.36034600	0.55638500
H	5.60942600	−3.85208400	−1.14413400
C	−7.01620900	0.81178000	0.10948000
H	−7.84684400	0.32774800	0.63950300
H	−6.74180500	1.70340800	0.69092300
H	−7.39194800	1.14674400	−0.86475100
C	−3.63977800	−1.56685200	−2.78036500
H	−2.62516400	−1.14929600	−2.87236200
H	−3.55553900	−2.64487300	−2.97379100
H	−4.25447200	−1.13101000	−3.57544900
C	−3.55466700	−2.14965700	2.23298600
H	−3.43414500	−3.23863200	2.14977800
H	−2.55262300	−1.73151900	2.41211300
H	−4.16179000	−1.94461000	3.12131600
H	−3.63954000	−4.54780700	−0.65460800
H	−1.04286200	−5.58994600	−0.71360200
C	−1.24049300	−2.33595600	−0.29348000
Ir	−0.55057900	−0.39568400	−0.07574400
H	−2.04752300	−0.08508100	0.16503200
H	−1.03699100	−0.15377900	−1.55152300
P	−0.26003900	1.95203200	−0.02302400
C	−1.86267900	2.80629600	0.22183800
C	−2.93937000	2.41743000	−0.58432400
C	−2.03735300	3.84091200	1.14357700
C	−4.16986600	3.04925600	−0.46354900
H	−2.81636200	1.61483000	−1.31363400
C	−3.27593900	4.46537300	1.26823200
H	−1.20551500	4.16112400	1.77046300
C	−4.34250400	4.07025700	0.46814900
H	−4.99693600	2.73819500	−1.10062200
H	−3.40446100	5.26571900	1.99452900
H	−5.30954300	4.56058800	0.56766300

C	0.40197800	2.72314300	−1.54031500
C	1.02947200	1.93843600	−2.51061300
C	0.30725400	4.10641800	−1.73308600
C	1.57847200	2.52655000	−3.64609000
H	1.07211800	0.85656000	−2.38029700
C	0.84866500	4.69191200	−2.87068300
H	−0.19502200	4.72572200	−0.98864500
C	1.48909200	3.90289400	−3.82430500
H	2.07340100	1.90824900	−4.39227800
H	0.76854300	5.76745300	−3.01611300
H	1.91374600	4.36391200	−4.71403600
C	0.80669500	2.57019400	1.33042200
C	1.94622200	3.34617600	1.10808100
C	0.48431300	2.18439100	2.63934000
C	2.74454400	3.73826600	2.18040700
H	2.21355800	3.64357600	0.09402000
C	1.28284500	2.57684700	3.70650600
H	−0.40750500	1.58149700	2.82235400
C	2.41568600	3.35697800	3.47714900
H	3.62519900	4.35295000	1.99886200
H	1.01927800	2.27626900	4.71891800
H	3.03783100	3.67083100	4.31349800
C	2.43871300	−0.32837900	1.48143300
C	3.56987200	0.30130100	1.98581500
C	4.50057400	0.80566700	1.08759500
C	4.31542900	0.65060800	−0.28983400
C	3.20101700	−0.04677100	−0.76224200
C	2.24505500	−0.52775000	0.11542700
H	3.67999600	0.43198400	3.05887900
H	5.36903300	1.33613600	1.47313300
H	3.11295000	−0.20683000	−1.83555100
H	1.46703100	−1.21546800	−0.26186700
C	5.25960200	1.20901800	−1.31869000
O	5.01716600	1.04836600	−2.49774100
C	6.46924500	1.96365800	−0.84659700
H	6.17829600	2.84150400	−0.25331900
H	7.10593200	1.33623300	−0.20846100
H	7.04465800	2.29258800	−1.71535900
N	1.43054900	−0.77426800	2.42953500
O	0.25370200	−0.89621100	2.03844500
O	1.76144200	−1.00068900	3.57277800

Substrate 14—Position 16 (Nitro Bound Transition State)

1 1

C	−1.45843500	−4.57539500	−0.60701000
C	−2.69215400	−4.08114300	−0.84988700
N	−0.64072600	−3.48850000	−0.33592400
N	−2.60675600	−2.70334200	−0.71736600
C	−3.73286100	−1.83239000	−0.91703800
C	−4.54841000	−1.52957200	0.17618100
C	−3.96676200	−1.33050900	−2.20146600
C	−5.62832500	−0.67607700	−0.04774300
C	−5.06176400	−0.48735600	−2.37270300
C	−5.90363600	−0.15170800	−1.31003400
H	−6.27520400	−0.41648300	0.79201300
H	−5.26717500	−0.08487600	−3.36624000
C	0.76583800	−3.60670700	−0.06589700
C	1.17765700	−3.92169000	1.23648900
C	1.66902900	−3.41875300	−1.11736600
C	2.54831500	−3.97795200	1.47885500
C	3.02942100	−3.48700100	−0.81871900
C	3.48729700	−3.74940700	0.47099600
H	2.89273300	−4.19576600	2.49168600
H	3.75290300	−3.32643300	−1.62048100
C	1.22067300	−3.13885600	−2.52095200
H	0.24623200	−3.59027800	−2.74744400
H	1.12954500	−2.05758000	−2.71331600
H	1.94918200	−3.52555300	−3.24280300
C	0.19061200	−4.19475700	2.33195600
H	−0.64468300	−3.48230600	2.32153400
H	−0.23707600	−5.20361200	2.24275000
H	0.67225100	−4.12602100	3.31372000
C	4.95484400	−3.80622900	0.76943400
H	5.17534500	−3.40399800	1.76664500
H	5.32580100	−4.84011300	0.75026300
H	5.53662200	−3.23560000	0.03521000
C	−7.08777800	0.73981000	−1.53483000
H	−7.51384000	1.09397400	−0.58801100
H	−6.82287200	1.61615300	−2.14123100
H	−7.88623100	0.21070900	−2.07280400
C	−3.06399700	−1.67324100	−3.34779600
H	−2.06392800	−1.23357800	−3.21380100
H	−2.92267700	−2.75787900	−3.45274600

H	−3.46891500	−1.29503200	−4.29272200
C	−4.25825300	−2.07511500	1.54082900
H	−4.17787800	−3.17091700	1.53880300
H	−3.30592300	−1.68549400	1.93239400
H	−5.04771900	−1.79937500	2.24804600
H	−3.62782400	−4.56266800	−1.10225500
H	−1.07078200	−5.58558900	−0.60175400
C	−1.34257400	−2.32702700	−0.39533400
Ir	−0.66342300	−0.38564300	−0.03400100
H	−2.21879900	−0.23123700	0.43509100
H	−1.32841900	0.08122400	−1.39231000
P	−0.61397200	1.97314200	0.44798200
C	−2.22633300	2.66505000	0.96333200
C	−3.38431500	2.25585000	0.29342000
C	−2.31329400	3.66605900	1.93587400
C	−4.60817100	2.84097000	0.59403400
H	−3.33545800	1.46819900	−0.46022400
C	−3.54349400	4.24010000	2.24045200
H	−1.42166500	3.99406800	2.46949200
C	−4.69070000	3.83103400	1.56910100
H	−5.50273200	2.51369600	0.06508500
H	−3.60373600	5.01075500	3.00645300
H	−5.65147600	4.28322400	1.80768300
C	−0.11617300	3.05482000	−0.93748100
C	0.10060600	2.54673800	−2.21916800
C	0.01089700	4.43276900	−0.71739100
C	0.46511400	3.39412500	−3.26126100
H	−0.02444900	1.48141000	−2.40671900
C	0.37114800	5.27712400	−1.75880400
H	−0.16962500	4.85001300	0.27354200
C	0.60350700	4.75762800	−3.03064500
H	0.64132000	2.98423700	−4.25361800
H	0.46869200	6.34586200	−1.57856700
H	0.88876800	5.42080100	−3.84489500
C	0.54333500	2.33143600	1.81104500
C	1.80372900	2.89045800	1.58133600
C	0.21962300	1.86955100	3.09433900
C	2.72323300	2.99065700	2.62101900
H	2.07554400	3.23744400	0.58466100
C	1.14167000	1.97175200	4.12840800
H	−0.75603700	1.41981900	3.28606500
C	2.39580700	2.53059600	3.89238700

H	3.70187800	3.42917400	2.43362300
H	0.88178800	1.60733400	5.12021500
H	3.11783400	2.60851700	4.70298100
C	3.39161800	−0.40502700	1.52909500
C	4.24569900	0.09417100	0.56691900
C	3.72332600	0.46482500	−0.68026500
C	2.36832100	0.31441800	−0.96197300
C	1.47017300	−0.19467200	−0.01947900
C	2.03538800	−0.53339600	1.22079700
H	3.73805800	−0.69106100	2.51962900
H	5.30624400	0.21335700	0.76980100
H	2.02955200	0.61197000	−1.95287800
H	0.33458600	−0.89228500	−1.18806300
N	1.16295800	−1.00173600	2.25778500
O	−0.07223400	−1.04261700	1.99727300
O	1.58519000	−1.33315600	3.34271700
C	4.57553800	1.04209400	−1.76390900
O	4.14411600	1.37820100	−2.84338900
O	5.85261100	1.14739400	−1.39464000
C	6.74581800	1.70794000	−2.37751900
H	6.36724300	2.69695800	−2.66470600
H	6.71619900	1.07447100	−3.27325100
C	8.11508300	1.76944100	−1.75783000
H	8.46242000	0.76999600	−1.47144300
H	8.83217200	2.18778700	−2.47291500
H	8.11423700	2.40378700	−0.86382600

Substrate 14—Position 17 (Nitro Bound C−H Activated Product)

1 1

C	−1.37648400	−4.40690800	0.21511500
C	−0.07724200	−4.63088000	0.50532500
N	−1.48898300	−3.06268000	−0.10918900
N	0.57679800	−3.41814700	0.35429400
C	1.98334100	−3.24948400	0.58934900
C	2.87108000	−3.49649400	−0.46242800
C	2.40982600	−2.81115000	1.84973800
C	4.22292500	−3.22632400	−0.24435800
C	3.76846900	−2.55693100	2.01528400
C	4.68706000	−2.74557500	0.97895200
H	4.93289500	−3.40172900	−1.05416700
H	4.12478800	−2.20771200	2.98617100

C	−2.76071500	−2.45115200	−0.37624600
C	−3.18897000	−2.32627400	−1.70134200
C	−3.54532000	−2.06329900	0.71629300
C	−4.42265000	−1.71514000	−1.91947100
C	−4.77173500	−1.46344700	0.44102500
C	−5.21898100	−1.26709600	−0.86591400
H	−4.76546500	−1.57744800	−2.94611000
H	−5.39251000	−1.12855000	1.27491900
C	−3.10873700	−2.30709500	2.13019100
H	−3.35115500	−3.33222200	2.44701100
H	−2.02691400	−2.17908700	2.26696400
H	−3.61556000	−1.62721800	2.82508300
C	−2.37033100	−2.84382600	−2.84563800
H	−1.37351700	−2.38559700	−2.88377600
H	−2.22688600	−3.93135200	−2.77239500
H	−2.86463300	−2.63558500	−3.80089200
C	−6.53324400	−0.59654700	−1.12839600
H	−6.53838300	−0.08818200	−2.10035300
H	−7.35819000	−1.32208300	−1.13735400
H	−6.76526200	0.14486300	−0.35271300
C	6.14122900	−2.45644700	1.19724600
H	6.72215600	−2.58260800	0.27634400
H	6.29495900	−1.43157700	1.56107800
H	6.56982400	−3.12831200	1.95279700
C	1.44693800	−2.62185100	2.98281300
H	0.72226400	−1.82052300	2.77629800
H	0.86283400	−3.53185000	3.17846800
H	1.97802900	−2.36144100	3.90485300
C	2.38887100	−4.00593000	−1.78737100
H	1.92015300	−4.99536700	−1.69694000
H	1.63303500	−3.34421900	−2.23722500
H	3.21796300	−4.09568000	−2.49728000
H	0.45572700	−5.52418600	0.80406200
H	−2.24136400	−5.05731600	0.20888000
C	−0.28804700	−2.43239300	−0.03296100
Ir	0.31613800	−0.43964200	−0.35009100
H	1.62923700	−1.04737800	−1.51094000
H	1.97528700	−1.17964100	−0.77501500
P	1.32866700	1.72761600	−0.37607900
C	2.91812800	1.74785500	−1.28583500
C	3.90570100	0.84686400	−0.86395200
C	3.19975100	2.63751000	−2.32310900

C	5.14550600	0.82594100	−1.48790900
H	3.71298900	0.16467900	−0.03065300
C	4.44473800	2.60975400	−2.94834100
H	2.45092500	3.36055900	−2.64416000
C	5.41434500	1.70369800	−2.53642100
H	5.90800900	0.12462100	−1.15267200
H	4.65464400	3.30516600	−3.75859800
H	6.38530400	1.68415700	−3.02739200
C	1.82616100	2.41263800	1.24517800
C	1.56255300	1.76417100	2.45213100
C	2.52146200	3.63014500	1.26145500
C	1.97196800	2.32638500	3.65862900
H	1.03498500	0.81237500	2.45319800
C	2.92628100	4.19043900	2.46488900
H	2.74748800	4.14119700	0.32458400
C	2.64994700	3.53935700	3.66578200
H	1.75370300	1.81376900	4.59340200
H	3.46520200	5.13581200	2.46673700
H	2.97030900	3.97879800	4.60847400
C	0.25264700	2.99542600	−1.12358900
C	−0.35674000	3.98132800	−0.34112500
C	−0.09416500	2.88231700	−2.47694700
C	−1.28598900	4.84805900	−0.90771600
H	−0.10922300	4.07258700	0.71583300
C	−1.02116800	3.75250100	−3.03807600
H	0.34562500	2.09647900	−3.09338500
C	−1.61931600	4.73560400	−2.25355900
H	−1.74998600	5.61642500	−0.29210500
H	−1.28447400	3.65389700	−4.08932400
H	−2.34657900	5.41509700	−2.69378800
C	−2.27031400	0.86626600	−0.60933100
C	−3.43663400	1.58268800	−0.34047900
C	−3.72458800	1.89288800	0.97455800
C	−2.86447200	1.45256200	1.99319600
C	−1.71212800	0.72944700	1.68472200
C	−1.35860900	0.42329700	0.36895100
H	−4.08319800	1.87980400	−1.16326600
H	−4.62220100	2.46131900	1.20956100
H	−1.09420400	0.40630100	2.52115900
H	1.00837200	−0.60195900	1.05227000
C	−3.14616000	1.70192500	3.44890300
O	−2.43616000	1.19921800	4.29782100

C	−4.31937700	2.56850100	3.81427400
H	−4.24176000	3.55999700	3.34889600
H	−5.26131900	2.12171700	3.46809900
H	−4.35606200	2.67858100	4.90088500
N	−1.97975500	0.53685300	−1.97500600
O	−0.90205700	−0.08187600	−2.19924500
O	−2.73151000	0.83225100	−2.87719500
